# Dopamine modulates antioxidant and phenolic responses to alleviate nickel stress in *Salvia officinalis*

**DOI:** 10.1186/s12870-026-08365-5

**Published:** 2026-02-11

**Authors:** Seyed Hamed Moazzami Farida, Nosrat Rahmani, Marzieh Taghizadeh, Benedicte Riber Albrectsen

**Affiliations:** 1 Department of Plant and Animal Biology, Faculty of Biological Science and Technology, University of Isfahan, Isfahan, Iran; 2 Department of Biology, Faculty of Science, Shahed University, Tehran, Iran; 3 Department of Plant Physiology, Umeå Plant Science Centre, Umeå University, Umeå, 90736 Sweden

**Keywords:** Antioxidant defense, Dopamine, Heavy metal, Nickel stress, Phenolic metabolism, *Salvia officinalis* l

## Abstract

**Background:**

Nickel (Ni) contamination is a significant constraint to agricultural sustainability and medicinal plant productivity, leading to oxidative stress, nutrient imbalance, and disruption of secondary metabolism. Dopamine (DA) has been reported as a stress-mitigating agent in plants. Still, its role in shaping antioxidants and phenolic responses to Ni toxicity in medicinal species, such as *Salvia officinalis*, remains poorly understood.

**Results:**

Exposure to increasing Ni concentrations (0–1000 µM) significantly reduced biomass (-46%), chlorophyll *b* (-57%), and shoot Ca and Fe contents (-50% and − 63%, respectively), while elevating oxidative markers (hydrogen peroxide (H_2_O_2_), malondialdehyde (MDA); 2.6-fold increase). Foliar DA application (0-100 µM) partially alleviated these effects by restoring biomass (+ 42%), enhancing Ca and Fe translocation (up to 1.7-fold), and maintaining carotenoid levels at nearly twice the control level under moderate stress. DA reduced oxidative markers by 16–24% and moderated the over-accumulation of superoxide dismutase (SOD), catalase (CAT), and peroxidase (POD) activities. Significantly, DA promoted phenolic-based antioxidant responses, increasing phenylalanine ammonia-lyase (PAL) (3.8-fold) and tyrosine aminotransferase (TAT) (1.5-fold) activities and stimulating rosmarinic acid accumulation (up to 91% above control). Multivariate analyses (principal component analysis (PCA), heatmap clustering, correlation networks, random forest) supported these findings, indicating that DA-treated plants clustered with low-stress phenotypes and shifted their defense balance toward phenolic rather than enzyme-dominated strategies.

**Conclusions:**

This study provides integrative physiological and metabolic evidence that DA enhances Ni tolerance in sage by reducing oxidative damage, supporting nutrient uptake, and reinforcing phenolic metabolism. These results highlight DA as a promising candidate biostimulant under controlled conditions, with relevance to the sustainable cultivation of medicinal plants in metal-contaminated soils, pending further validation.

**Supplementary Information:**

The online version contains supplementary material available at 10.1186/s12870-026-08365-5.

## Background

Heavy metal pollution, particularly nickel (Ni), is one of the most well-known environmental threats, with adverse effects on ecosystem stability, soil integrity, agricultural sustainability, and plant productivity [1]. In Iran, sources of Ni contamination extend beyond industrial activities to include agricultural practices such as wastewater irrigation and fertilizer application, as well as proximity to industrial areas. These human-driven activities have contributed to increased Ni concentrations in farm soils, posing a significant challenge [2]. While Ni plays an essential role at trace levels, excessive Ni accumulation induces severe physiological disorders in plants, including impaired photosynthesis, ionic imbalance, disrupted nutrient uptake, and excessive production of reactive oxygen species (ROS), ultimately leading to oxidative stress and membrane damage [3, 4].

To cope with oxidative stress, plants rely on an intricate defense system comprising enzymatic antioxidants, such as superoxide dismutase (SOD), peroxidase (POD), and catalase (CAT), as well as non-enzymatic antioxidants, including phenolics and flavonoids; however, under intense or prolonged metal exposure, these intrinsic systems may become insufficient. A variety of elicitors, including salicylic acid, nitric oxide donors, calcium, and melatonin, have been investigated for their ability to mitigate metal-induced oxidative stress by modulating antioxidant capacity [5–8]. While these compounds predominantly stimulate enzymatic responses, non-enzymatic antioxidant pathways, particularly those associated with phenolic metabolism, remain comparatively underrepresented in the literature. Moreover, elicitor effectiveness is highly variable across plant species, concentrations, and exposure durations [9], underscoring the need to identify compounds with broader and more consistent regulatory potential across multiple defense layers.

Dopamine (DA), a biogenic catecholamine traditionally recognized as a neurotransmitter in animals, is also endogenously synthesized in a wide range of plant species, where it functions as a phenolic antioxidant involved in various physiological processes, including responses to environmental stress [10]. Recent studies have demonstrated that exogenous application of DA can mitigate the negative effects of abiotic stressors such as salinity, drought, and heavy metal toxicity. These protective effects are mainly attributed to DA’s antioxidant capacity, including ROS scavenging, enhancement of antioxidant enzyme activities, and metal-chelating properties that reduce heavy metal toxicity [11–13]. Application methods of DA include root treatment, supplementation in hydroponic nutrient solutions, and foliar spraying, all of which have been shown to improve growth, membrane stability, and chlorophyll retention under stress conditions in species such as apple, tomato, and cabbage [12, 14, 15]. Although the precise signaling mechanisms of DA in plants remain under investigation, its role in coordinating multiple layers of stress defense, particularly under heavy metal toxicity, remains poorly understood.

*Salvia officinalis* L. (common sage) is a widely used medicinal and aromatic plant, frequently employed as a reference species in studies on secondary metabolism and abiotic stress physiology [16–18], and its cultivation in semi-arid regions may be affected by heavy metal contamination of soils, which is increasingly prevalent in such environments [2]. Its rich accumulation of rosmarinic acid (RA) provides a unique framework for exploring phenolic-based defense mechanisms under Ni stress. With high phenolic levels central to antioxidant capacity and marked sensitivity to environmental fluctuations, sage represents an appropriate model for dissecting biochemical stress responses [18]. Despite extensive research on sage under abiotic stresses, no study has systematically examined whether DA can simultaneously modulate ionomic balance and phenolic metabolism in this species under Ni toxicity. Addressing this gap is particularly important in light of the increasing incidence of soil metal contamination and the dual agronomic and pharmacological value of sage.

While DA has been associated with stress mitigation in several plant species, its integrative role in coordinating ionomic balance, oxidative regulation, and phenolic metabolism under Ni toxicity in medicinal plants remains largely unexplored. The primary objective of this study was to elucidate how DA modulates *S. officinalis* response*s* to Ni stress. We hypothesized that DA alleviates Ni-induced damage not only by reducing oxidative stress but also by reorienting antioxidant defense toward phenolic-based regulation. This shift has not been systematically reported under heavy metal stress.

## Materials and methods

### Plant material and growth conditions

*S. officinalis* seeds were obtained from the Medicinal Plants Garden, Ferdowsi University of Mashhad (Iran). Seeds were surface-sterilized with 1% (v/v) sodium hypochlorite for 2 min, rinsed thoroughly, and subjected to cold stratification at 4 °C for 7 days. Germinated seedlings were transplanted into 12.5 × 14 cm plastic pots filled with garden soil (pH 7.4, EC 1.2 dS/m), coco peat, and perlite (3:1:1 v/v). Plants were grown in a greenhouse under a 16-hour light/8-hour dark photoperiod (600 µmol/m^2^/s), with a relative humidity of 65 ± 5% and a temperature range of 22–25 °C. Pots were randomly repositioned every five days.

### Experimental design

Two weeks post-transplantation, the plants were irrigated every other day with 100 mL of distilled water. From week 3, Ni stress was imposed by applying NiSO_4_·6H_2_O (Merck, Germany) at 0, 50, 100, 250, 500, and 1000 µM Ni^2+^ twice weekly (100 mL per pot). This concentration range was selected to encompass mild, moderate, and severe stress levels in *S. officinalis*, allowing for the evaluation of both regulated physiological responses and phytotoxic effects. The 1000 µM concentration represents a threshold-level stress rather than an environmentally realistic exposure. After two weeks of Ni treatment, foliar dopamine (DA) hydrochloride (≥ 98% purity; Sigma-Aldrich, USA) was applied at 0, 50, and 100 µM (50 mL per plant) in the early morning. Foliar applications were made gradually with a fine-mist sprayer until uniform leaf wetting was achieved, avoiding runoff or stomatal flooding. Ni and DA treatments were each applied twice weekly on alternating days. The experiment was arranged as a completely randomized factorial design with two fixed factors (3 DA × 6 Ni), and each treatment combination included three biological replicates (one plant per pot). Plants were harvested 60 days after transplantation for physiological, biochemical, and elemental analyses.

### Morphological indexes

The harvested plants were separated into roots and shoots. The fresh weight (FW) of the aerial parts was immediately measured in grams (g). Subsequently, the samples were oven-dried at 60 ℃ for 48 h, and the dry weight (DW) was recorded using a precision balance.

### Physiological and biochemical measurements

#### Photosynthetic pigments

Photosynthetic pigments were extracted from 100 mg FW of leaves (control and Ni/DA-treated) using 80% (v/v) acetone. Chlorophyll *a* (Chl *a*), chlorophyll *b* (Chl *b*), and total carotenoid contents were quantified spectrophotometrically at 647, 663, and 470 nm, respectively (UV/Vis spectrophotometer, Perkin Elmer, USA), following the method described by Lichtenthaler [19]. Pigment concentrations were expressed as mg/g FW.

#### Nutrient content

Root and shoot samples were analyzed for essential elements to assess the nutrient composition of *S. officinalis* plants. After harvest, the samples were thoroughly rinsed with distilled water to remove surface contaminants. They were then dried in an oven at 60 °C to obtain a constant dry weight. Subsequently, 5 mL of 1 M nitric acid (HNO_3_) and 2 mL of H_2_O_2_ were added to 0.5 g of dried root and shoot tissues, respectively. The mixtures were then heated at 95 °C for 3 h to ensure complete digestion. The digested solutions were filtered through a 0.45 μm pore-size filter. The concentrations of calcium (Ca), potassium (K), magnesium (Mg), iron (Fe), manganese (Mn), and Ni were quantified by an inductively coupled plasma mass Spectrometry (ICP-MS; ELAN DRC-e, Perkin Elmer, USA). Results were calculated against calibration standards and expressed as mg/g DW.

To evaluate the efficiency of nutrient mobility from roots to aerial parts, the translocation factor (TF) was calculated for each element using the following formula:

$$\:TF=\frac{Element\:concentration\:in\:Shoot\:\left(\frac{mg}{g}DW\right)}{Element\:concentration\:in\:Root\:\left(\frac{mg}{g}DW\right)}$$

This ratio reflects the plant’s relative capacity to transport absorbed ions from the root system to above-ground tissues under different treatment conditions.

#### Total soluble proteins and enzymes assay

Total soluble proteins (TSP) were extracted from 250 mg of frozen leaf tissue using 1 mL of chilled phosphate buffer (100 mM, pH 6.0), supplemented with 2 mM EDTA, 4 mM DTT, and 2% (w/w) polyvinylpyrrolidone. This process was performed using a mortar and pestle on ice for 15 min. The resulting homogenate was then centrifuged at 13,000 × g for 25 min at 4 °C to separate the clear supernatant, which was subsequently stored at − 80 °C for future use. Protein quantification was performed using the Bradford method [20], employing bovine serum albumin as a standard, and results were expressed as mg/g FW.

The activity of peroxidase (POD, EC 1.11.1.7) was determined by monitoring guaiacol oxidation, following the method described by Abeles & Biles [21]. Catalase (CAT, EC 1.11.1.6) activity was assayed by measuring the decomposition rate of hydrogen peroxide (H_2_O_2_) as described by Cakmak & Horst [22]. Superoxide dismutase (SOD, EC 1.15.1.1) activity was evaluated based on its ability to inhibit the photoreduction of nitroblue tetrazolium (NBT), as described in the protocol by Giannopolitis & Ries [23]. All enzymatic activities were standardized and reported in the common unit µkat/mg protein.

The activity of L-phenylalanine ammonia-lyase (PAL, EC 4.3.1.5) in leaf tissues was determined spectrophotometrically by measuring the formation of trans-cinnamic acid, following the method described by Heide et al. [24]. Tyrosine aminotransferase (TAT, EC 2.6.1.5) activity was evaluated according to Diamondstone [25], using the molar absorptivity of 4-hydroxybenzaldehyde (24,900 L/mol/cm) to quantify product formation. Both enzyme activities were standardized and expressed in nkat/mg protein.

#### MDA, H_2_O_2,_ and proline contents

Malondialdehyde (MDA), an indicator of lipid membrane damage, was quantified using the protocol established by Heath & Packer [26]. The results are expressed as µM/g FW.

To assess hydrogen peroxide (H_2_O_2_) accumulation, we used the potassium iodide assay as described by Sergiev et al., [27], with concentrations also reported in µM/g fresh weight (FW).

Proline levels in leaf samples subjected to treatment were determined using a colourimetric reaction with ninhydrin, as described by Bates [28]. A calibration graph was created using a range of standard proline concentrations (0–2 mg/L), and the measured values were reported as µmol/g FW.

#### Phenolic compound

To extract phenolic compounds, 1 g of air-dried ground plant tissue was suspended in 5 mL of methanol. This mixture was incubated at room temperature (25 °C) for 24 h with constant shaking. After incubation, it was centrifuged at 12,000 rpm for 10 min, and the supernatant was collected for analysis.

The total phenolic content (TPC) in the extract was evaluated using the Folin-Ciocalteu method, as described by Singleton et al. [29]. Quantitative estimation was conducted using a standard curve created from gallic acid (GAE) solutions ranging from 0 to 0.5 mg/L, with absorbance measurements recorded at 750 nm. The phenolic content was reported as mg GAE/g DW.

The total flavonoid content (TFC) was determined using an aluminum chloride colorimetric assay based on the method described by Zhishen et al. [30]. Quercetin (QE) was used to generate a calibration curve at 415 nm, and the results were expressed as mg QE/g DW.

#### HPLC of polyphenols

High-performance liquid chromatography (HPLC) was performed on a Smartline HPLC system (Agilent 1200 series, Germany) equipped with a C18 reverse-phase column (5 μm, 150 × 4.6 mm) maintained at 25 °C. The mobile phase consisted of solvent A (HPLC-grade methanol) and solvent B (1% v/v aqueous formic acid), using the following gradient: 10% A at 0 min, increased to 25% A at 10 min, 60% A at 25 min, and held at 70% A between 30 and 40 min. The flow rate was 1 mL/min with an injection volume of 20 µL. UV detection was performed at 280 nm. Samples were filtered through 0.22 μm syringe filters before injection. Polyphenols were identified by comparing retention times with those of authentic standards and quantified using compound-specific calibration curves.

### Statistical analysis

Statistical analyses were performed using SPSS (v26). Data were analyzed using a completely randomized factorial design with Ni and DA as fixed factors. A fixed-effect two-way ANOVA was used to assess the main and interactive effects of Ni and DA. When significant differences were detected (*p* < 0.05), one-way ANOVA followed by Tukey’s HSD test was used for pairwise comparisons. Data were presented as means ± SD (tables) or means ± SE (figures).

Multivariate analyses, including PCA and hierarchical heatmap clustering, were performed in R (v4.3.2; https://www.r-project.org/) within the RStudio interface to explore trait variation and treatment grouping. Pearson correlation coefficients (|r| ≥ 0.85) were calculated in SPSS after verifying normality, and the resulting correlation-based interaction networks were visualized in Cytoscape (v3.10.1) as a complementary visualization to integrate and summarize strong trait-trait relationships observed across treatments. In parallel, Random Forest (RF) analysis was conducted using MetaboAnalyst 6.0 (https://www.metaboanalyst.ca) as an exploratory, hypothesis-generating approach to qualitatively assess trait contributions to treatment discrimination. RF outputs, including Mean Decrease Accuracy (MDA), were used solely for descriptive purposes rather than for statistically validated variable-importance ranking, given the limited sample size and high dataset dimensionality.

## Results

A two-way ANOVA was performed to assess the individual and interactive effects of Ni stress and DA application on physiological, biochemical, and antioxidant traits in *S. officinalis*. As detailed in Supplementary Table S1, both factors exerted statistically significant main effects across all measured parameters (Ni: *p* < 0.001; DA: *p* < 0.001), with several significant interactions also observed (Ni × DA: *p* < 0.05). Where appropriate, one-way ANOVA followed by Tukey’s HSD test was applied to compare treatment means. These statistical approaches provided a robust basis for the trait-wise interpretation presented in the following sections.

### DA partially offset Ni-induced biomass reduction in S. officinalis

Ni exposure significantly decreased (*p* < 0.05) biomass production in *S. officinalis*, with both FW and DW exhibiting progressive decreases in response to increasing Ni concentrations (FW: Ni main effect, *p* < 0.001; DW: Ni main effect, *p* < 0.001) (Table 1). In untreated plants (D0), FW declined from 5.17 g at 0 µM Ni to 2.79 g at 1000 µM Ni (− 46%), while DW decreased from 0.60 g to 0.34 g over the same gradient. Foliar application of 100 µM DA (D100) partially mitigated this growth suppression, increasing FW and DW to 3.97 g and 0.46 g, respectively, at 1000 µM Ni, corresponding to relative increases of ~ 42% and 35% compared to D0 (under identical stress conditions). This mitigation was supported by a significant Ni × DA interaction for FW (*p* = 0.049), whereas the interaction effect was not significant for DW (*p* > 0.05). At intermediate and non-stressed conditions, DA treatment also improved biomass, albeit to a lesser extent, with increases of ~ 7% in FW and DW for unstressed plants.

**Table 1:** Effect of nickel (Ni, µM) stress and dopamine (DA, µM) treatment on shoot fresh weight (FW), dry weight (DW), and photosynthetic pigments (chlorophyll *a*, chlorophyll *b*, and carotenoids) in *S. officinalis*. Data represent means ± SD (*n* = 3). Different lowercase letters indicate statistically significant differences among treatments, as determined by tukey’s HSD test (*p* < 0.05)

Samples		FW (g)	DW (g)	Chlorophyll a (mg/g FW)	Chlorophyll b (mg/g FW)	Carotenoids (mg/g FW)
Dopamine 0	Ni 0	5.17 ± 0.26^ab^	0.60 ± 0.03^ab^	1.49 ± 0.23^a−d^	0.53 ± 0.05^a−c^	0.36 ± 0.06^b−d^
	Ni 50	4.84 ± 0.16^a−e^	0.56 ± 0.02^a−d^	1.18 ± 0.24^c−g^	0.45 ± 0.05^cd^	0.31 ± 0.03^cd^
	Ni 100	4.29 ± 0.28^b−g^	0.50 ± 0.03^b−f^	1.04 ± 0.10^d−h^	0.41 ± 0.02^d−f^	0.44 ± 0.06^a−d^
	Ni 250	4.13 ± 0.16^d−g^	0.49 ± 0.03 ^c−f^	0.93 ± 0.06^e−h^	0.34 ± 0.03^fg^	0.55 ± 0.06^a−d^
	Ni 500	3.53 ± 0.20^gh^	0.42 ± 0.03^fg^	0.77 ± 0.16^gh^	0.29 ± 0.03^gh^	0.25 ± 0.05^d^
	Ni 1000	2.79 ± 0.29^h^	0.34 ± 0.03^g^	0.60 ± 0.18^h^	0.23 ± 0.02^h^	0.25 ± 0.04^d^
Dopamine 50	Ni 0	4.90 ± 0.62^a−d^	0.57 ± 0.07^a−d^	1.77 ± 0.09^ab^	0.55 ± 0.06^ab^	0.37 ± 0.07^b−d^
	Ni 50	4.88 ± 0.20^a−d^	0.56 ± 0.02^a−d^	1.55 ± 0.16^a−c^	0.49 ± 0.03^b−d^	0.42 ± 0.05^a−d^
	Ni 100	4.71 ± 0.48^a−f^	0.54 ± 0.06^a−e^	1.41 ± 0.21^b−e^	0.45 ± 0.03^c−e^	0.39 ± 0.05^b−d^
	Ni 250	4.49 ± 0.28^b−f^	0.52 ± 0.03^b−f^	1.18 ± 0.08^c−g^	0.44 ± 0.03^c−e^	0.71 ± 0.04^a^
	Ni 500	4.09 ± 0.19^d−g^	0.47 ± 0.02^d−f^	0.85 ± 0.07^gh^	0.35 ± 0.03^e−g^	0.30 ± 0.04^cd^
	Ni 1000	3.86 ± 0.09^fg^	0.45 ± 0.01^ef^	0.64 ± 0.15^h^	0.34 ± 0.04^fg^	0.37 ± 0.05^b−b^
Dopamine 100	Ni 0	5.53 ± 0.17^a^	0.64 ± 0.02^a^	1.90 ± 0.06^ab^	0.59 ± 0.02^a^	0.31 ± 0.18^cd^
	Ni 50	5.12 ± 0.24^a−c^	0.59 ± 0.03^a−c^	1.92 ± 0.13^a^	0.51 ± 0.03^a−d^	0.32 ± 0.09^cd^
	Ni 100	4.89 ± 0.23^a−d^	0.56 ± 0.04^a−d^	1.74 ± 0.15^ab^	0.49 ± 0.02^a−d^	0.49 ± 0.30^a−d^
	Ni 250	4.54 ± 0.32^b−f^	0.52 ± 0.05^b−f^	1.40 ± 0.30^b−f^	0.46 ± 0.03^b−d^	0.64 ± 0.06^ab^
	Ni 500	4.26 ± 0.37^c−g^	0.49 ± 0.04^b−f^	0.91 ± 0.07^f−h^	0.42 ± 0.04^d−f^	0.48 ± 0.07^a−d^
	Ni 1000	3.97 ± 0.23^e−g^	0.46 ± 0.03^d−f^	0.67 ± 0.23^h^	0.33 ± 0.03^fg^	0.59 ± 0.09^a−c^

### Differential response of photosynthetic pigments to Ni and DA treatments

Ni toxicity significantly altered the photosynthetic pigment composition in *S. officinalis*, with Chl *b* and carotenoids being exceptionally responsive, while Chl *a* remained largely unaffected across treatments (Chl *a*: Ni, *p* = 0.533; DA, *p* = 0.638) (Table 1). In DA-untreated plants (D0), increasing Ni concentrations induced a progressive decline in Chl *b* content, which dropped by 56.6%, from 0.53 mg/g FW at 0 µM Ni to 0.23 mg/g FW at 1000 µM Ni. This response was supported by significant main effects of Ni (*p* < 0.001) and DA (*p* < 0.001) on Chl *b* content. DA application significantly attenuated this decline in a concentration-dependent manner. For instance, under 250 µM Ni, treatment with 100 µM DA (D100) restored Chl *b* levels by approximately 35% relative to the corresponding D0 group. At 500 µM Ni, Chl *b* content in D100-treated plants reached 0.42 mg/g FW, representing a 1.45-fold increase compared to the corresponding untreated group (D0N500).

Carotenoid levels also displayed notable sensitivity to both Ni toxicity and DA modulation. While Ni alone led to a gradual decrease in carotenoid content in D0 plants (from 0.36 to 0.25 mg/g FW), DA exerted a strong stimulatory effect. This response was supported by significant main effects of Ni (*p* < 0.001) and DA (*p* = 0.007), as well as a significant Ni × DA interaction (*p* = 0.020), on carotenoid content. The most prominent enhancement was observed at 250 µM Ni with 50 µM DA, where carotenoid content peaked at 0.71 mg/g FW, corresponding to approximately a two-fold increase relative to the control and representing the highest value among all treatments (Table 1). Even at the highest Ni level (1000 µM), carotenoid concentrations in DA-treated plants remained significantly higher than in the corresponding untreated group.

### Ni strongly affects nutrient accumulation and translocation

To assess the impact of Ni and DA on nutrient dynamics in *S. officinalis*, the concentrations of essential macro- (Ca, K, Mg) and micronutrients (Fe, Mn, Ni) were quantified in both root and shoot tissues. Their root-to-shoot translocation efficiencies were evaluated using translocation factors (TFs) (Tables 2 and 3). Ni exposure markedly impaired the uptake and mobility of Ca (Ni main effect: *p* < 0.001). Shoot Ca amount decreased from 21.06 mg/g DW in control plants to 12.46 mg/g at 500 µM Ni and further to 10.98 mg/g at 1000 µM, reflecting a nearly twofold reduction. Root Ca also declined in parallel, by ~ 50%. This suppression was mirrored by a moderate decrease in Ca-TF under severe stress (4.6% reduction relative to the control at 1000 µM Ni), indicating that both uptake and translocation were adversely affected. Based on the results obtained, the DA application partially restored Ca homeostasis under Ni stress. Compared to D0N500, shoot Ca content in D100N500 plants increased by 44.6%, and Ca-TF rose to 13.43, exceeding the values observed in both the Ni-stressed treatment (D0N500, 12.46) and the DA-alone treatment (D100N0, 12.80), although the Ni × DA interaction for Ca-related traits was not significant (*p* = 0.074).

**Table 2:** Effect of nickel (Ni, µM) stress and dopamine (DA, µM) treatment on macro- and micronutrient contents [Ca, K, Mg, Fe, Mn, and Ni] in shoots and roots of *S. officinalis*. Data represent means ± SD (*n* = 3). Different lowercase letters indicate statistically significant differences among treatments, as determined by tukey’s HSD test (*p* < 0.05)

Samples		Shoot (mg/g DW)						Root (mg/g DW)						
		Ca	K	Mg	Fe	Mn	Ni	Ca	K	Fe	Mn	Mg	Ni	
Dopamine 0	Ni 0	21.06 ± 2.74^a^	31.50 ± 0.92^ab^	0.28 ± 0.03^a^	0.27 ± 0.05^a^	0.17 ± 0.02^ab^	0.00 ± 0.00^g^	1.61 ± 0.12^a^	4.45 ± 0.12^a^	1.21 ± 0.13^a^	0.66 ± 0.06^a^	0.03 ± 0.00^a-c^	0.00 ± 0.00^g^	
	Ni 50	21.30 ± 8.77^a^	28.73 ± 1.35^a-c^	0.25 ± 0.04^f^	0.24 ± 0.02^e^	0.13 ± 0.02^i^	0.02 ± 0.01^fg^	1.46 ± 0.09^a-c^	4.25 ± 0.09^ab^	1.08 ± 0.03^a-d^	0.52 ± 0.04^a-c^	0.03 ± 0.00^d-f^	0.20 ± 0.10f^g^	
	Ni 100	18.61 ± 2.05^ab^	25.52 ± 1.21^c-f^	0.22 ± 0.03^a^	0.23 ± 0.02^a^	0.12 ± 0.02^a-e^	0.11 ± 0.00^e-g^	1.32 ± 0.24^a-e^	3.45 ± 0.24^c-e^	0.97 ± 0.08^a-d^	0.48 ± 0.05^a-e^	0.03 ± 0.00^e-g^	0.97 ± 0.03^e-g^	
	Ni 250	16.45 ± 5.88^a-c^	22.36 ± 0.65^e-h^	0.20 ± 0.03^ef^	0.20 ± 0.03^de^	0.11 ± 0.02^hi^	0.26 ± 0.03^de^	1.14 ± 0.39^c-f^	3.06 ± 0.39^d-f^	0.90 ± 0.09^a-e^	0.45 ± 0.03^c-f^	0.02 ± 0.00f^g^	2.42 ± 0.26^de^	
	Ni 500	12.46 ± 5.00^c-e^	21.34 ± 1.07^f-h^	0.17 ± 0.02^ab^	0.12 ± 0.03^a^	0.10 ± 0.01^a-c^	0.52 ± 0.06^c^	1.00 ± 0.31^d-f^	2.84 ± 0.31^ef^	0.84 ± 0.11^de^	0.38 ± 0.03^d-f^	0.02 ± 0.00^gh^	4.79 ± 0.59^cd^	
	Ni 1000	10.98 ± 1.53d^e^	18.98 ± 1.28^h^	0.15 ± 0.02^f^	0.10 ± 0.02^ed^	0.08 ± 0.01^f-i^	0.94 ± 0.18^a^	0.81 ± 0.29^f^	2.55 ± 0.29^f^	0.66 ± 0.10^e^	0.30 ± 0.06^f^	0.02 ± 0.00^h^	7.73 ± 1.08^a^	
Dopamine 50	Ni 0	19.77 ± 2.78^a^	31.74 ± 1.19^a^	0.28 ± 0.05^a-c^	0.27 ± 0.00^a^	0.15 ± 0.02^a-f^	0.00 ± 0.00^g^	1.64 ± 0.11^ab^	4.59 ± 0.11^a^	1.14 ± 0.08^a^	0.65 ± 0.06^ab^	0.03 ± 0.01^ab^	0.00 ± 0.00^g^	
	Ni 50	20.37 ± 3.43^ab^	28.57 ± 1.65^a-c^	0.26 ± 0.02^d-f^	0.24 ± 0.02^de^	0.14 ± 0.02^g-i^	0.02 ± 0.01^fg^	1.62 ± 0.14^a-c^	4.24 ± 0.14^ab^	1.13 ± 0.05^a-c^	0.59 ± 0.02^a-c^	0.03 ± 0.00^b-d^	0.20 ± 0.05^fg^	
	Ni 100	21.03 ± 2.78^a^	26.37 ± 1.11^b-e^	0.28 ± 0.01^a-c^	0.25 ± 0.02^a^	0.13 ± 0.01^a-d^	0.08 ± 0.01^fg^	1.65 ± 0.05^a^	3.85 ± 0.05^a-c^	1.05 ± 0.05^a-d^	0.54 ± 0.03^a^	0.03 ± 0.00^c-e^	0.73 ± 0.10^fg^	
	Ni 250	17.16 ± 3.13^ab^	25.97 ± 0.82^c-f^	0.23 ± 0.01^b-f^	0.23 ± 0.02^cd^	0.13 ± 0.02^e-h^	0.18 ± 0.02^ef^	1.38 ± 0.05^a-d^	3.68 ± 0.05^b-d^	1.00 ± 0.05^a-d^	0.51 ± 0.02^a-d^	0.03 ± 0.00^d-f^	1.63 ± 0.23^ef^	
	Ni 500	15.77 ± 2.80^b-d^	21.38 ± 2.69^f-h^	0.21 ± 0.01^ab^	0.17 ± 0.02^a^	0.11 ± 0.03^ab^	0.42 ± 0.03^cd^	1.28 ± 0.35^b-f^	2.97 ± 0.35^d-f^	0.84 ± 0.12^b-e^	0.45 ± 0.01^b-f^	0.03 ± 0.00^fg^	3.85 ± 0.31^cd^	
	Ni 1000	11.39 ± 2.47^de^	19.54 ± 0.85^gh^	0.15 ± 0.01^a-c^	0.12 ± 0.02^bc^	0.07 ± 0.00^b-f^	0.86 ± 0.05^ab^	0.72 ± 0.38^ef^	2.56 ± 0.38^f^	0.71 ± 0.12^e^	0.34 ± 0.03^ef^	0.02 ± 0.00^h^	5.69 ± 0.75^ab^	
Dopamine 100	Ni 0	21.22 ± 6.23^a^	30.73 ± 1.05^a^	0.28 ± 0.01^a-e^	0.28 ± 0.01^ab^	0.18 ± 0.02^d-g^	0.00 ± 0.00^g^	1.66 ± 0.21^a^	4.52 ± 0.21^a^	1.16 ± 0.02^a^	0.70 ± 0.02^a^	0.03 ± 0.00^a^	0.00 ± 0.00^g^	
	Ni 50	19.60 ± 5.06^a^	30.36 ± 1.06^ab^	0.27 ± 0.01^c-f^	0.26 ± 0.01^bc^	0.16 ± 0.01^e-h^	0.01 ± 0.01^g^	1.64 ± 0.14^ab^	4.35 ± 0.14^ab^	1.14 ± 0.05^ab^	0.64 ± 0.05^ab^	0.03 ± 0.00^a-c^	0.10 ± 0.05^g^	
	Ni 100	20.34 ± 3.51^a^	29.06 ± 1.20^a-c^	0.27 ± 0.01^ab^	0.24 ± 0.03^a^	0.16 ± 0.01^a^	0.06 ± 0.02^fg^	1.67 ± 0.18^ab^	4.21 ± 0.18^ab^	1.07 ± 0.03^a-d^	0.59 ± 0.03^ab^	0.03 ± 0.00^b-d^	0.55 ± 0.21^fg^	
	Ni 250	19.39 ± 4.49^ab^	28.38 ± 2.17^b-d^	0.25 ± 0.02^a-d^	0.25 ± 0.03^ab^	0.13 ± 0.02^c-g^	0.14 ± 0.02^e-g^	1.45 ± 0.27^a-c^	4.04 ± 0.27^a-c^	1.01 ± 0.03^a-d^	0.52 ± 0.01^a-c^	0.03 ± 0.00^d-f^	1.31 ± 0.19^e-g^	
	Ni 500	18.01 ± 4.17^ab^	24.00 ± 1.86^d-g^	0.24 ± 0.02^a^	0.20 ± 0.03^a^	0.12 ± 0.02^a^	0.35 ± 0.03^d^	1.34 ± 0.32^a-c^	3.41 ± 0.32^c-e^	0.83 ± 0.11^c-e^	0.48 ± 0.01^a-c^	0.03 ± 0.00^ef^	3.26 ± 0.25^de^	
	Ni 1000	11.22 ± 3.06^e^	20.25 ± 1.92^f-h^	0.14 ± 0.01^a-c^	0.13 ± 0.02^a^	0.10 ± 0.02^a-e^	0.71 ± 0.09^b^	0.92 ± 0.31^f^	2.88 ± 0.31^ef^	0.67 ± 0.11^e^	0.34 ± 0.04^f^	0.02 ± 0.00^h^	5.93 ± 0.41^b^	

*officinalis*. Data represent means ± SD (n = 3). Different lowercase letters indicate statistically significant differences among treatments, as determined by Tukey’s HSD test (*p* < 0.05)

**Table 3:** Effect of nickel (Ni, µM) stress and dopamine (DA, µM) treatment on nutrient translocation factors (TF) for Ca, K, Mg, Fe, Mn, and Ni in *S. officinalis*. TF values were calculated as the ratio of the nutrient concentration in shoots to that in roots and are presented as derived indices calculated from statistically analyzed elemental concentration data

Samples		Ca	K	Mg	Fe	Mn	Ni
Dopamine 0	Ni 0	13.06	7.07	0.42	0.22	4.78	0.00
	Ni 50	14.54	6.77	0.48	0.22	4.05	0.11
	Ni 100	14.11	7.39	0.47	0.23	4.17	0.11
	Ni 250	14.44	7.30	0.44	0.23	4.37	0.11
	Ni 500	12.46	7.50	0.44	0.14	4.55	0.11
	Ni 1000	13.48	7.45	0.49	0.15	4.19	0.12
Dopamine 50	Ni 0	12.07	6.91	0.42	0.24	4.46	0.00
	Ni 50	12.55	6.74	0.45	0.21	4.33	0.11
	Ni 100	12.76	6.85	0.52	0.24	3.76	0.11
	Ni 250	12.40	7.05	0.46	0.23	4.29	0.11
	Ni 500	12.37	7.20	0.46	0.20	4.22	0.11
	Ni 1000	15.83	7.64	0.46	0.17	3.74	0.15
Dopamine 100	Ni 0	12.80	6.81	0.40	0.24	5.06	0.00
	Ni 50	11.98	6.98	0.43	0.22	4.69	0.11
	Ni 100	12.20	6.90	0.46	0.23	4.57	0.11
	Ni 250	13.40	7.02	0.49	0.25	4.16	0.11
	Ni 500	13.43	7.04	0.50	0.24	4.09	0.11
	Ni 1000	12.18	7.03	0.42	0.20	5.72	0.12

Iron accumulation progressively decreased with rising Ni concentrations (Ni main effect on shoot and root Fe: *p* < 0.001). In the control plants, the shoot and root Fe contents were 0.27 and 1.21 mg/g DW, respectively. At 1000 µM Ni, shoot Fe was reduced by approximately 63%, while root Fe declined by 45%. This was accompanied by a 32% decrease in Fe-TF, which dropped from 0.22 to 0.15 (Ni effect on Fe-TF: *p* < 0.001). The DA application alleviated these reductions (DA main effect on shoot Fe: *p* < 0.001). In D100N250 and D100N500 plants, shoot Fe concentrations increased to 0.25 and 0.20 mg/g DW, representing ~ 1.25- and 1.67-fold increases relative to their corresponding Ni-only treatments. Root Fe levels remained relatively unchanged (1.01 and 0.83 mg/g DW, respectively; DA effect on root Fe: *p* = 0.123, not significant). Fe-TF values also improved, rising from 0.23 to 0.25 in D100N250 (~ 9% increase) and from 0.14 to 0.24 in D100N500, indicating a 1.7-fold enhancement in Fe mobility to the shoot, despite the absence of a significant Ni × DA interaction (*p* = 0.104).

Among other elements, K and Mg concentrations showed moderate to high reductions under Ni stress (Ni main effects: *p* < 0.001). Still, their translocation remained relatively stable, as K-TF and Mg-TF values exhibited only minor fluctuations (Ni × DA interaction: *p* > 0.05). In contrast, Mn levels and TFs varied inconsistently, with no discernible dose-dependent trend, and DA application had little impact on Mn behavior. These results indicate that DA improved the accumulation and translocation efficiency of Ca and Fe under Ni stress while having limited effects on Mn.

### Oxidative stress and antioxidants show distinct responses

#### DA efficiently counteracts Ni-induced H_2_O_2_ and MDA accumulation

**Fig. 1:**
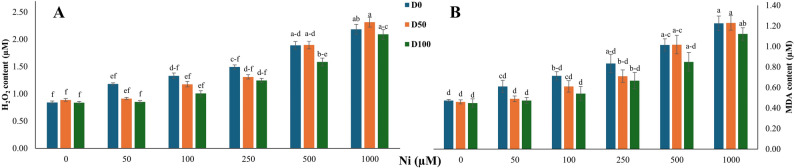
Effect of nickel (Ni) stress and dopamine (DA) on oxidative stress markers in *S. officinalis*. **A** Hydrogen peroxide (H_2_O_2_) content and (**B**) malondialdehyde (MDA) content under different Ni and DA treatments. Data represent means ± SE (*n* = 3). Different lowercase letters indicate statistically significant differences among treatments, as determined by Tukey’s HSD test (*p* < 0.05)

Ni exposure caused a dose-dependent increase in oxidative stress biomarkers in *S. officinalis* (Ni main effect: *p* < 0.001). In DA-untreated plants (D0), H_2_O_2_ content increased 2.6-fold at 1000 µM Ni relative to 0 µM Ni (Fig. 1A), while MDA levels exhibited a similar 2.6-fold increase (Fig. 1B). Exogenous DA application reduced oxidative markers under Ni stress (DA main effect: *p* = 0.017 for H_2_O_2_; *p* = 0.056 for MDA).

DA application resulted in modest but statistically significant reductions in H_2_O_2_ (4.2%) and MDA (8.2%) levels under severe Ni stress. Under 250 µM Ni, D100 further decreased H_2_O_2_ and MDA by 16.6% and 21.1%, respectively. At 100 µM Ni, both markers exhibited reductions of approximately 24%. All reported changes were statistically significant. The Ni × DA interaction was not significant for either H_2_O_2_ or MDA (*p* > 0.05).

#### DA modulates antioxidant enzyme activities under Ni stress

**Fig. 2:**
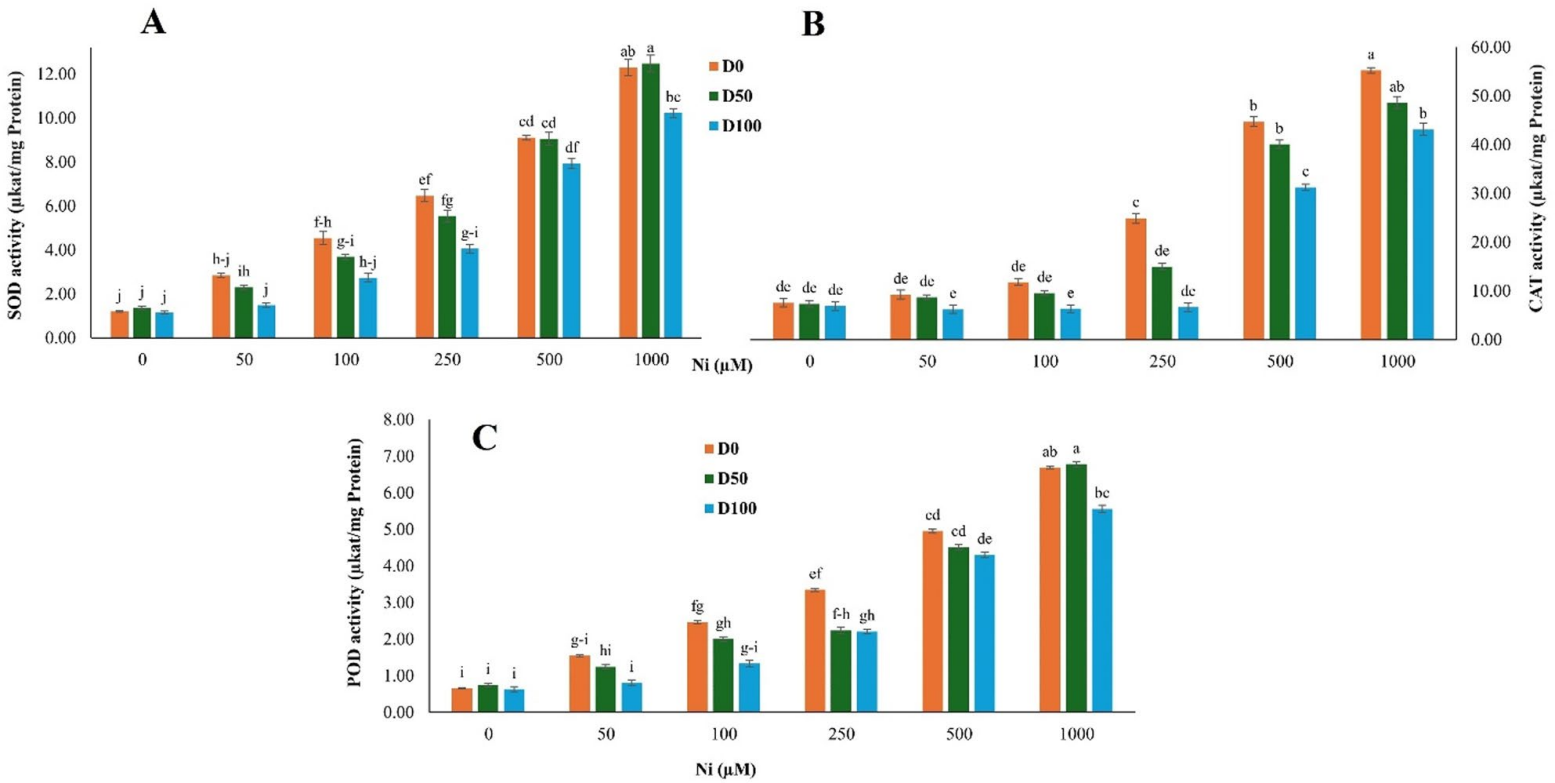
Effect of nickel (Ni) stress and dopamine (DA) on antioxidant enzyme activities in *S. officinalis*. **A** Superoxide dismutase (SOD), (**B**) catalase (CAT), and (**C**) peroxidase (POD) activities under different Ni and DA treatments. Data represent means ± SE (*n* = 3). Different lowercase letters indicate statistically significant differences among treatments, as determined by Tukey’s HSD test (*p* < 0.05)

Ni exposure induced a concentration-dependent increase in the activities of SOD, CAT, and POD in *S. officinalis* (Ni main effect: *p* < 0.001 for all enzymes; Fig. 2A–C). SOD showed the strongest and most consistent response, increasing from 1.21 µkat/mg protein in control plants to 6.47 and 9.11 µkat/mg at 250 and 500 µM Ni, respectively, and peaking at 12.30 µkat/mg under 1000 µM Ni, an approximately 10-fold rise (Fig. 2A). CAT activity showed a strong Ni-dependent induction, increasing up to 7.3-fold at 1000 µM Ni relative to the control (Fig. 2B). POD activity increased progressively along the Ni concentration gradient, reaching an approximately 10.1-fold increase at 1000 µM Ni, with a slower rate of induction between 250 and 500 µM Ni (Fig. 2C). Exogenous DA (100 µM) significantly modulated these enzymatic responses (DA main effect: *p* < 0.001 for SOD, CAT, and POD).

At 250 µM Ni, DA reduced SOD, CAT, and POD activities by 37.2%, 73.3%, and 34.1%, respectively. Under 1000 µM Ni, DA application led to decreases of 16.8% in SOD, 21.9% in CAT, and 17.0% in POD compared to Ni-only plants. A significant Ni × DA interaction was observed only for CAT activity (*p* < 0.001), whereas interactions for SOD and POD were not significant (*p* > 0.05).

#### DA alters non-enzymatic antioxidant responses under Ni stress

**Fig. 3:**
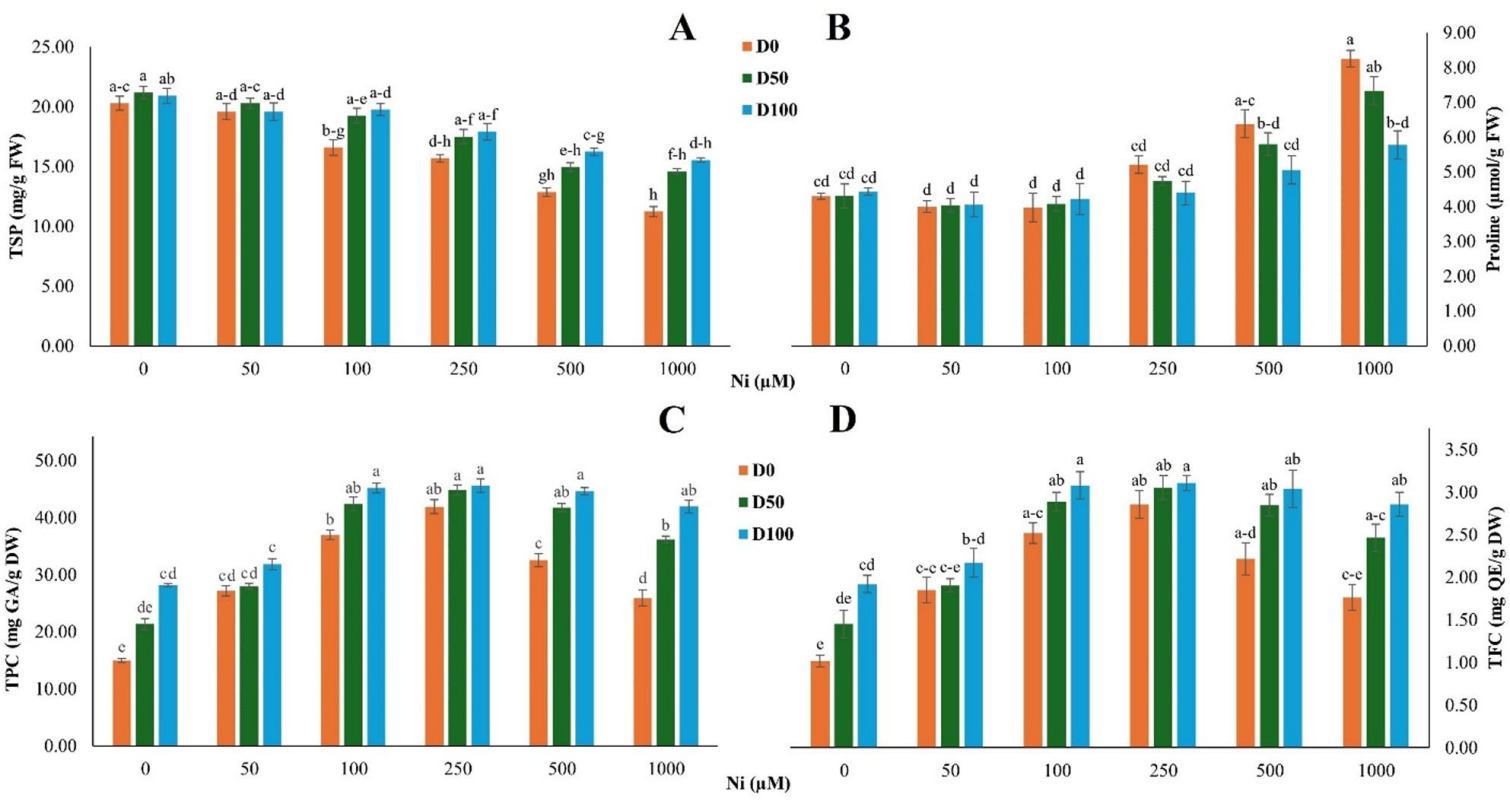
Effect of nickel (Ni) stress and dopamine (DA) on non-enzymatic antioxidant traits in *S. officinalis*. **A** Total soluble protein (TSP), (**B**) proline content, (**C**) total phenolic content (TPC), and (**D**) total flavonoid content (TFC) under different Ni and DA treatments. Data represent means ± SE (*n* = 3). Different lowercase letters indicate statistically significant differences among treatments, as determined by Tukey’s HSD test (*p* < 0.05)

Non-enzymatic antioxidant responses in *S. officinalis* under Ni stress exhibited compound-specific trends (Fig. 3). TSP decreased progressively with increasing Ni concentrations (Ni main effect: *p* < 0.001), with the lowest value recorded at 1000 µM Ni (20.28 mg/g FW), representing an approximate 50% reduction compared to the control. Application of 100 µM DA significantly increased TSP under severe Ni stress (DA main effect: *p* < 0.001), partially restoring protein levels relative to the Ni-only treatment (Fig. 3A), although the Ni × DA interaction was not significant (*p* = 0.455).

Proline content remained stable under mild Ni exposure (up to 100 µM), ranging between 4.0 and 4.4 µmol/g FW. At higher Ni levels, proline accumulation was strongly induced (Ni main effect: *p* < 0.001), reaching 8.25 µmol/g FW at 1000 µM Ni in untreated plants. DA application significantly attenuated proline accumulation (DA main effect: *p* = 0.016), resulting in an approximately 30% reduction relative to the Ni-only treatment at 1000 µM Ni. Similarly, at 500 µM Ni, DA application reduced proline content by approximately 21%, with no significant Ni × DA interaction (*p* = 0.080) (Fig. 3B).

In contrast, secondary antioxidants were markedly enhanced by Ni stress. TPC increased significantly with rising Ni concentration (Ni main effect: *p* < 0.001), showing an approximately 2.8-fold increase at 250 µM Ni in untreated plants (Fig. 3C). DA application further enhanced TPC levels (DA main effect: *p* < 0.001), increasing TPC to 45.61 mg GAE/g DW under the same conditions. At 1000 µM Ni, TPC remained higher in DA-treated plants (41.94 mg/g DW) than in untreated ones (25.92 mg/g DW), with a significant Ni × DA interaction (*p* < 0.001). TFC showed a similar pattern. TFC increased significantly under Ni stress (Ni main effect: *p* < 0.001) and was further promoted by DA application (DA main effect: *p* < 0.001; Ni × DA interaction: *p* < 0.001). At 250 µM Ni, TFC in DA-treated plants exhibited an approximately threefold increase relative to the corresponding baseline level (Fig. 3D).

#### DA influences PAL and TAT activity under Ni stress

**Fig. 4:**
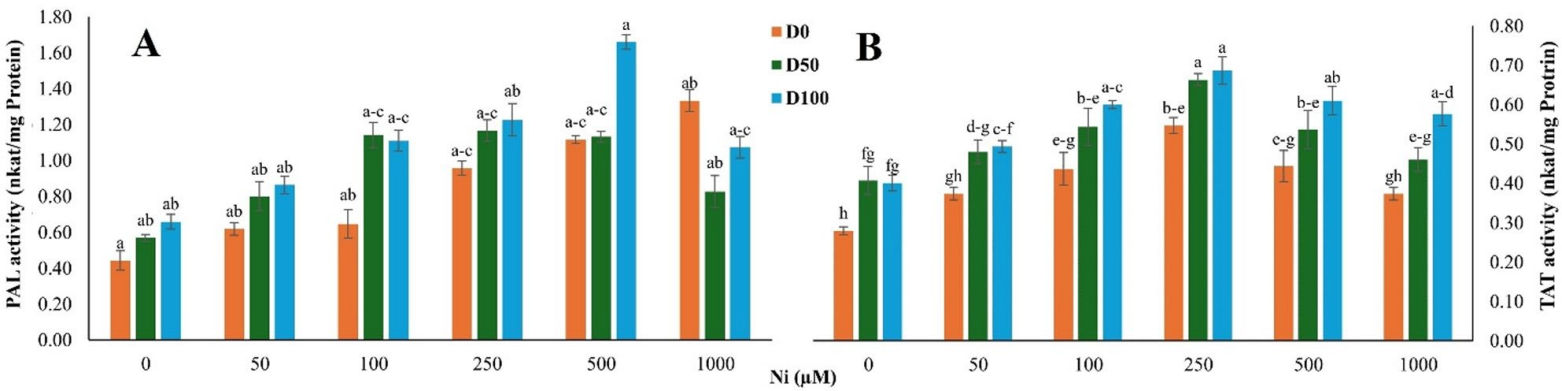
Effect of nickel (Ni) stress and dopamine (DA) on key enzymes of the phenylpropanoid pathway in *S. officinalis*. **A** Phenylalanine ammonia-lyase (PAL) activity and (**B**) tyrosine aminotransferase (TAT) activity under different Ni and DA treatments. Data represent means ± SE (*n* = 3). Different lowercase letters indicate statistically significant differences among treatments, as determined by Tukey’s HSD test (*p* < 0.05)

Ni stress and DA application significantly affected PAL and TAT activities. These two key enzymes initiate and regulate the phenylpropanoid pathway responsible for the biosynthesis of phenolic antioxidants (Ni main effect: *p* < 0.001 for both enzymes; DA main effect: *p* = 0.019 for PAL and *p* < 0.001 for TAT; PAL: Fig. 4A; TAT: Fig. 4B). PAL activity increased progressively with rising Ni concentrations in untreated plants, reaching an approximately 3.0-fold increase at 1000 µM Ni relative to the control (Fig. 4A). DA application (100 µM) alone elevated PAL activity by approximately 1.5-fold under non-stress conditions. Under combined treatments, the highest PAL activity was observed at 500 µM Ni in DA-treated plants, corresponding to an approximately 3.8-fold increase relative to the control (Fig. 4A). Even under severe stress (1000 µM Ni), PAL activity in DA-treated plants remained elevated compared to Ni-only treatments. The Ni × DA interaction for PAL was not statistically significant (*p* = 0.093).

TAT activity followed a similar but less pronounced pattern. DA alone increased TAT activity by approximately 1.4-fold, while Ni stress induced a maximum response at 250 µM Ni in untreated plants (Fig. 4B). Under the same conditions, DA-treated plants showed approximately a 1.5-fold increase in TAT activity compared with the control. At 1000 µM Ni, TAT activity declined but remained higher in DA-treated plants than in untreated ones. No significant Ni × DA interaction was detected for TAT (*p* = 0.292).

### Phenolic compound profiling by HPLC reveals DA-induced changes under Ni stress in S. officinalis

HPLC-based phenolic profiling revealed that gallic acid (GA) and rosmarinic acid (RA) were the most responsive compounds to Ni stress and DA application in *S. officinalis* (Table 4). In untreated plants, GA content initially decreased by approximately 27% under moderate Ni stress (250 µM Ni), followed by a marked increase at 1000 µM Ni, exceeding control levels by ~ 20% (Ni main effect: *p* < 0.001). In contrast, RA levels increased almost linearly with rising Ni concentrations, reaching a maximum at 500 µM Ni with an approximately 91% increase relative to the control, followed by a slight decline at 1000 µM Ni (Ni main effect: *p* < 0.001).

**Table 4:** Effect of nickel (Ni, µM) stress and dopamine (DA, µM) treatment on phenolic compounds quantified by HPLC in *S. officinalis*. Phenolic contents are expressed as mg/g DW. Data represent means ± SD (*n* = 3). Different lowercase letters indicate statistically significant differences among treatments, as determined by tukey’s HSD test (*p* < 0.05)

Samples		Gallic Acid (mg/g DW)	Catechin (mg/g DW)	Quercetin (mg/g DW)	Vanillin (mg/g DW)	Ferulic Acid (mg/g DW)	Rosmarinic Acid (mg/g DW)	Chlorogenic Acid (mg/g DW)
Dopamine 0	Ni 0	1.01 ± 0.1^d−g^	0.31 ± 0.01^a−c^	0.96 ± 0.01^b^	0.07 ± 0.01^b−d^	0.41 ± 0.05^a^	5.08 ± 0.42^ij^	0.20 ± 0.02^cd^
	Ni 50	0.93 ± 0.06^d−g^	0.32 ± 0.01^ab^	1.14 ± 0.01^a^	0.06 ± 0.01^b−d^	0.13 ± 0.01^b^	6.70 ± 0.25^fg^	0.15 ± 0.01^c−e^
	Ni 100	0.84 ± 0.08^d−g^	0.25 ± 0.01^d−g^	0.93 ± 0.01^bc^	0.04 ± 0.01^b−d^	0.15 ± 0.01^b^	8.74 ± 0.25^a−c^	0.10 ± 0.01^ed^
	Ni 250	0.74 ± 0.06^fg^	0.26 ± 0.02^c−g^	0.96 ± 0.05^b^	0.03 ± 0.01^d^	0.16 ± 0.01^b^	8.90 ± 0.51^ab^	0.14 ± 0.01^ed^
	Ni 500	0.78 ± 0.04^e−g^	0.29 ± 0.01^a−d^	0.86 ± 0.04^b−e^	0.04 ± 0.01^b−d^	0.18 ± 0.01^b^	9.72 ± 0.31^a^	0.17 ± 0.02^c−e^
	Ni 1000	1.21 ± 0.07^cd^	0.27 ± 0.01^b−e^	0.70 ± 0.03^d−h^	0.03 ± 0.01^d^	0.16 ± 0.01^b^	8.15 ± 0.26^v−e^	0.13 ± 0.01^ed^
Dopamine 50	Ni 0	0.77 ± 0.09^e−g^	0.22 ± 0.02^gh^	0.63 ± 0.02^gh^	0.02 ± 0.01^d^	0.15 ± 0.01^b^	5.12 ± 0.28^ij^	0.11 ± 0.01^ed^
	Ni 50	0.88 ± 0.03^d−g^	0.20 ± 0.01^h^	0.58 ± 0.02^h^	0.03 ± 0.01^cd^	0.14 ± 0.01^b^	5.32 ± 0.37^h−j^	0.12 ± 0.01^ed^
	Ni 100	1.19 ± 0.06^cd^	0.22 ± 0.01^gh^	0.64 ± 0.01^gh^	0.04 ± 0.01^b−d^	0.13 ± 0.01^b^	6.16 ± 0.27^f−i^	0.14 ± 0.01^ed^
	Ni 250	1.14 ± 0.01^c−e^	0.25 ± 0.01^d−g^	0.69 ± 0.06^e−h^	0.03 ± 0.01^cd^	0.15 ± 0.02^b^	7.26 ± 0.55^d−f^	0.13 ± 0.01^ed^
	Ni 500	0.91 ± 0.03^d−g^	0.26 ± 0.01^d−g^	0.76 ± 0.02^c−g^	0.04 ± 0.01^cd^	0.16 ± 0.01^b^	8.58 ± 0.55^a−d^	0.11 ± 0.01^ed^
	Ni 1000	0.65 ± 0.03^g^	0.23 ± 0.02^e−h^	0.64 ± 0.06^gh^	0.03 ± 0.01^d^	0.16 ± 0.01^b^	7.48 ± 0.56^v−f^	0.09 ± 0.04^e^
Dopamine 100	Ni 0	2.07 ± 0.03^a^	0.23 ± 0.01^e−h^	0.65 ± 0.01^f−h^	0.04 ± 0.01^cd^	0.17 ± 0.01^b^	4.65 ± 0.48^j^	0.20 ± 0.03^cd^
	Ni 50	1.67 ± 0.04^b^	0.22 ± 0.01^f−h^	0.72 ± 0.02^d−h^	0.07 ± 0.01^b−d^	0.14 ± 0.02^b^	5.31 ± 0.27^h−j^	0.25 ± 0.02^bc^
	Ni 100	1.40 ± 0.11^bc^	0.23 ± 0.03^e−h^	0.86 ± 0.03^b−e^	0.09 ± 0.02^ab^	0.09 ± 0.01^b^	5.41 ± 0.36^g−i^	0.39 ± 0.05^a^
	Ni 250	1.20 ± 0.02^cd^	0.28 ± 0.06^a−d^	0.84 ± 0.01^b−e^	0.08 ± 0.01^a−c^	0.15 ± 0.01^b^	6.24 ± 0.43^f−i^	0.31 ± 0.04^ab^
	Ni 500	1.07 ± 0.04^c−f^	0.32 ± 0.02^a^	0.87 ± 0.02^b−d^	0.13 ± 0.02^a^	0.20 ± 0.01^b^	7.09 ± 0.89^ef^	0.10 ± 0.02^ed^
	Ni 1000	0.92 ± 0.03^d−f^	0.27 ± 0.01^c−f^	0.81 ± 0.02^b−f^	0.13 ± 0.02^a^	0.16 ± 0.02^b^	6.63 ± 1.55^f−h^	0.08 ± 0.01^e^

statistically significant differences among treatments, asdetermined by Tukey’s HSD test (*p* < 0.05)

DA application altered these patterns in a dose-dependent manner (DA main effect: *p* < 0.001 for both GA and RA). At 50 µM DA, GA content increased to levels comparable to a ~ 1.2-fold enhancement under mild-to-moderate Ni stress, while RA content exhibited an approximately 1.4-fold increase at 500 µM Ni. At Ni concentrations below 1000 µM, DA-treated plants maintained higher RA levels (approximately 30–40% higher) compared to untreated plants. At 100 µM DA, GA content peaked at approximately a twofold level relative to the control in the absence of Ni, whereas RA continued to increase, reaching a maximum at 500 µM Ni. Other phenolic compounds, including catechin, quercetin, chlorogenic acid, and vanillin, showed moderate or relatively stable trends, and DA treatment mainly maintained them rather than inducing them strongly under Ni stress. Statistically significant effects of Ni and DA were primarily confined to GA and RA (*p* < 0.05), whereas changes in other phenolic compounds were moderate or non-significant (Table 4).

### Multivariate analysis reveals response patterns under Ni stress and DA treatment

To comprehensively characterize the multidimensional responses of *S. officinalis* to Ni toxicity and DA supplementation, we applied a suite of multivariate statistical approaches. Given the interrelated nature of physiological, biochemical, and ionomic traits under abiotic stress, univariate analyses alone were insufficient to capture the complexity of plant responses. Therefore, we employed principal component analysis (PCA), hierarchical clustering, correlation heatmaps, network inference, and random forest classification to uncover treatment-specific response patterns, co-regulated trait clusters, and the most discriminative features across experimental groups.

#### PCA highlights Ni-driven separation and DA-mediated modulation of physiological and biochemical traits

**Fig. 5:**
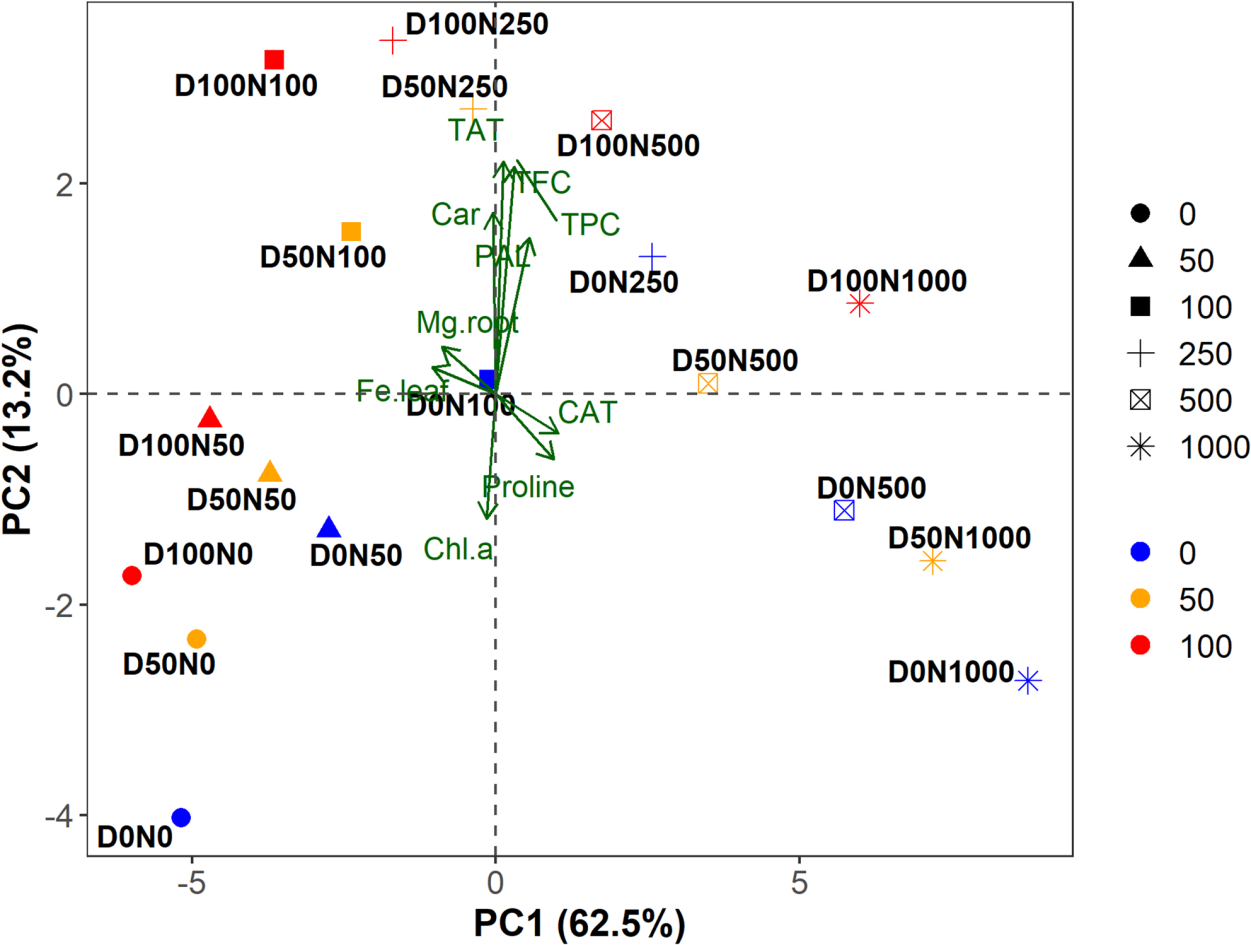
Principal component analysis (PCA) biplot illustrating the separation of treatments and associated trait loadings in S. officinalis under nickel (Ni) stress and dopamine (DA) treatments. Treatments were coded as D0, D50, and D100 (0, 50, and 100 µM DA) combined with Ni levels (N0–N1000). PC1 and PC2 explain 62.5% and 13.2% of the total variance, respectively. Symbols represent Ni levels, while colors denote DA treatments. Trait vectors (green arrows) highlight major contributors to treatment separation, including antioxidant enzymes, phenolic metabolism, pigments, and nutrient traits. Trait abbreviations: Chl *a*, chlorophyll *a*; Car, carotenoids; CAT, catalase; PAL, phenylalanine ammonia-lyase; TAT, tyrosine aminotransferase; TPC, total phenolic content; TFC, total flavonoid content; Mg, magnesium; Fe, iron

PCA effectively distinguished the treatment groups, with PC1 and PC2 explaining 62.5% and 13.2% of the variance, respectively, accounting for a cumulative 75.7% of the total variability (Fig. 5). Along PC1, the strongest separation was observed between low and high Ni levels, indicating that Ni stress was the dominant factor driving divergence among treatments. PC2 further differentiated DA-treated samples, particularly those receiving 100 µM DA under moderate Ni stress, which clustered closely with PAL, TAT, TPC, and TFC, reflecting enhanced antioxidant and phenolic responses. By contrast, control and low-stress plants grouped with traits such as Chl *a*, Mg in the root, and Fe in the leaf, indicative of a preserved basal physiological status.

MANOVA revealed significant main effects of DA (Pillai’s trace = 0.84, F = 34.52, *p* < 0.001) and Ni (Pillai’s trace = 0.97, F = 195.72, *p* < 0.001), while their interaction was not significant (*p* = 0.382). Chi-square and Monte Carlo analyses confirmed that sample separation was primarily driven by Ni (χ^2^ = 34.6, *p* = 0.0028), with DA exerting a smaller but consistent effect (*p* = 0.0108).

#### Hierarchical heatmap clustering highlights trait-specific coordination under Ni stress and DA supplementation

**Fig. 6:**
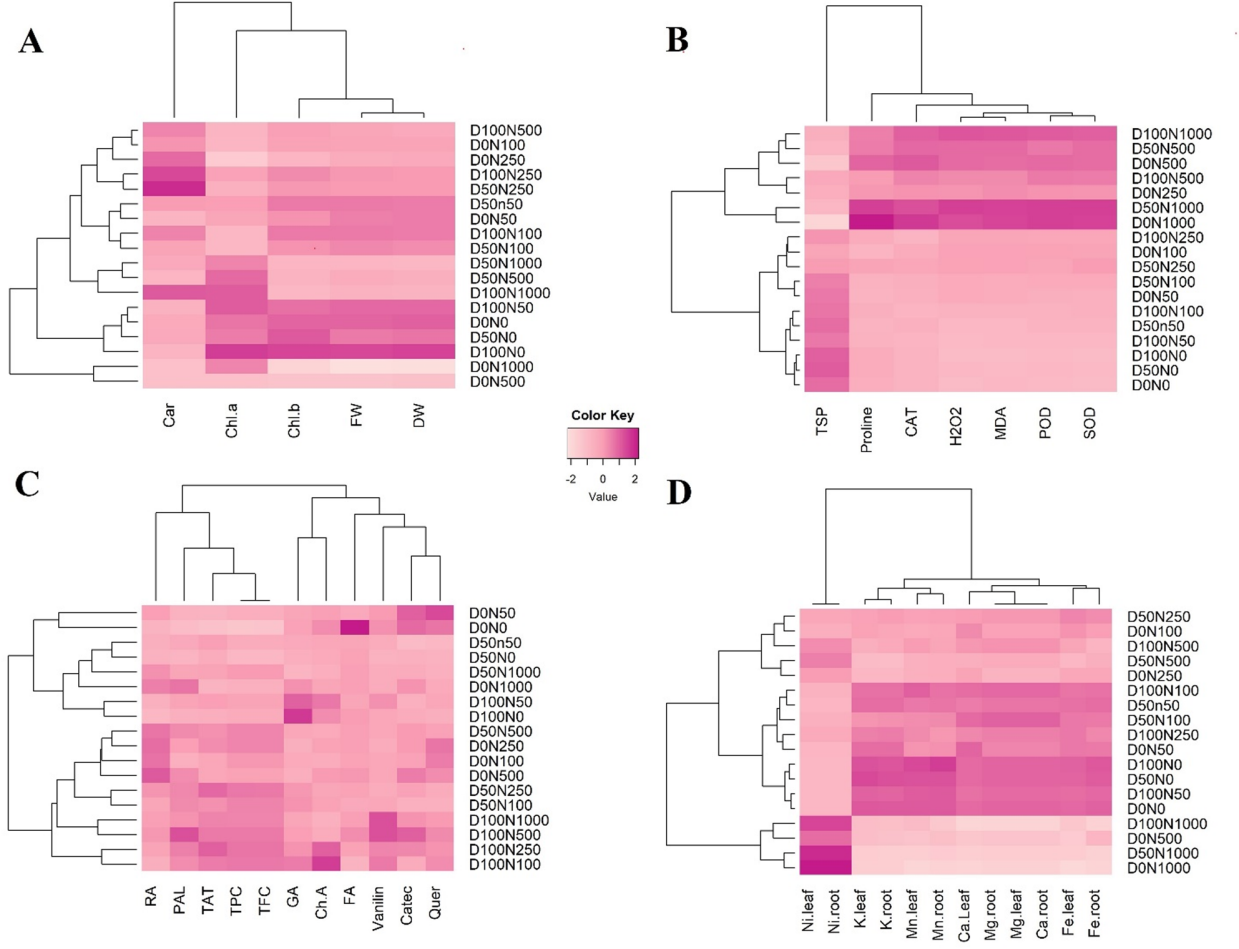
Hierarchical clustering heatmaps showing trait-specific responses of *S. officinalis* under nickel (Ni) stress and dopamine (DA) treatments. Panel **A **illustrates growth and pigment traits; panel **B **displays oxidative stress markers and antioxidant enzymes; panel **C **presents phenolic metabolism traits and individual phenolic compounds; and panel **D **shows macro- and micronutrient profiles in root and leaf tissues. Treatments were coded as D0, D50, and D100 (0, 50, and 100 µM DA) combined with Ni levels (N0–N1000). The color scale represents normalized Z-scores (− 2 to + 2), where darker shades indicate higher values relative to the mean. Trait abbreviations: FW, fresh weight; DW, dry weight; Chl *a*, chlorophyll *a*; Chl *b*, chlorophyll *b*; Car, carotenoids; H_2_O_2_, hydrogen peroxide; MDA, malondialdehyde; TSP, total soluble protein; SOD, superoxide dismutase; CAT, catalase; POD, peroxidase; PAL, phenylalanine ammonia-lyase; TAT, tyrosine aminotransferase; TPC, total phenolic content; TFC, total flavonoid content; GA, gallic acid; RA, rosmarinic acid; QE, quercetin; FA, ferulic acid; Catec, catechin; ChA, chlorogenic acid; Ca, calcium; K, potassium; Mg, magnesium; Fe, iron; Mn, manganese; Ni, nickel

Hierarchical heatmap clustering was applied across four functional datasets to unravel treatment-driven response patterns in *S. officinalis* under differential Ni stress and DA supplementation (Fig. 6A–D). This multi-layered approach enabled the visualization of co-regulated traits and segregation of treatments based on stress severity and DA-mediated modulation.

In the growth, pigment, and biochemical datasets (Fig. 6A–B), high Ni treatments (≥ 500 µM) clustered together, showing pronounced declines in FW, DW, Chl *b*, and carotenoids, alongside elevated oxidative markers (H_2_O_2_, MDA, and proline) and heightened antioxidant enzyme activities (SOD, POD, and CAT), indicative of severe stress. In contrast, Chl *a* declined less sharply, and carotenoids were better preserved in DA-treated plants, particularly under low to moderate Ni stress. DA supplementation shifted several treatments closer to the control cluster, characterized by improved pigment retention, reduced oxidative markers, balanced antioxidant enzyme activity, and increased TSP levels.

Similarly, phenolic and enzymatic traits (Fig. 6C) were separated into three clusters. Cluster I included high Ni without DA (D0N1000, D0N500) and D50N1000, characterized by elevated RA, PAL activity, and catechin levels. Cluster II comprised moderate Ni treatments (D0N100, D0N250, D50N250) and DA-supplemented samples under moderate to high Ni (D50N500, D100N250, D100N500, D100N1000), showing higher vanillin and chlorogenic acid along with moderate TAT and gallic acid. Cluster III contained control and low-Ni samples (D0N0, D0N50, D50N0, D50N50, D100N0, D100N50), associated with increased ferulic acid and lower overall phenolic accumulation. Distinct clustering patterns were observed for phenolic compounds and enzyme activities across Ni levels and DA treatments.

For ion distribution (Fig. 6D), the clustering analysis revealed three major groups distinguished by Ni intensity and nutrient status. The first group, representing high Ni exposure (D0N1000, D50N1000, D100N1000, D0N500), displayed strong Ni accumulation in both roots and leaves, accompanied by reductions in K, Ca, Mg, and Mn, indicating nutrient displacement under severe Ni stress. The second group encompassed low to moderate Ni treatments, characterized by enriched levels of K, Ca, Mg, and Mn in both tissues, alongside comparatively lower Ni levels. The third group included treatments with Ni levels ranging from moderate to relatively high, characterized by intermediate Ni levels and other nutrient values. This stratification highlights distinct differences in ionic balance among treatments, which align with Ni severity and DA application.

#### The correlation matrix and network topology reveal coordinated stress modules under Ni toxicity

**Fig. 7:**
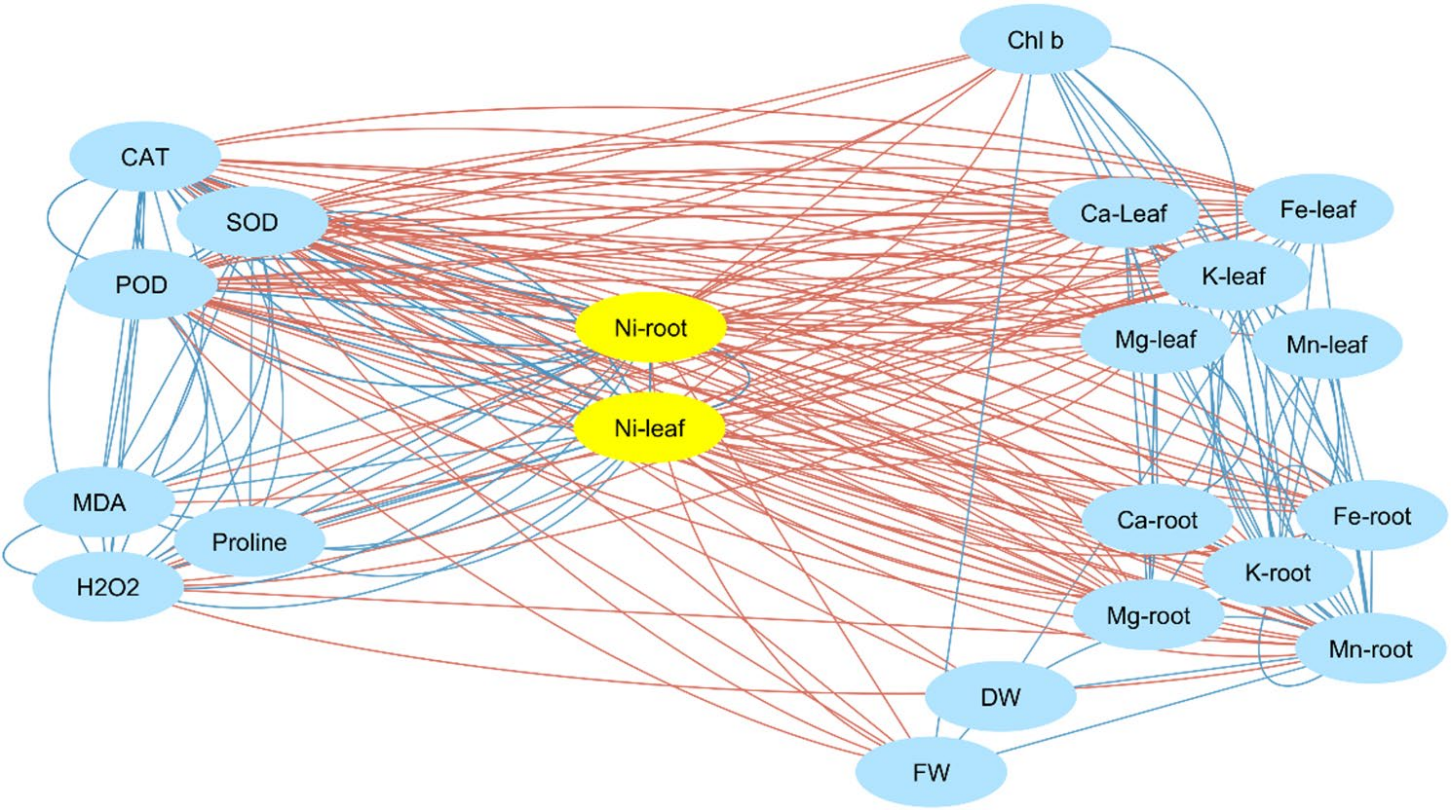
Correlation network analysis illustrating the interrelationships among physiological, biochemical, and nutrient traits in *S. officinalis* under nickel (Ni) stress and dopamine (DA) treatments. Nodes represent individual traits, with blue lines denoting positive correlations and red lines indicating negative correlations (|r| ≥ 0.85, *p* < 0.05). Ni-root and Ni-leaf (yellow) act as central hubs negatively associated with nutrient traits, while strong positive links are observed among growth (FW, DW), pigments (Chl *b*), and nutrient-related variables (Ca, K, Mg, Fe, Mn in leaf and root tissues). Antioxidant and oxidative stress markers (SOD, CAT, POD, MDA, H_2_O_2_, proline) form a separate cluster linked to Ni accumulation, highlighting distinct functional modules under stress conditions. Trait abbreviations: FW, fresh weight; DW, dry weight; Chl *b*, chlorophyll *b*; H_2_O_2_, hydrogen peroxide; MDA, malondialdehyde; SOD, superoxide dismutase; CAT, catalase; POD, peroxidase; Ca, calcium; K, potassium; Mg, magnesium; Fe, iron; Mn, manganese; Ni-leaf and Ni-root, nickel in leaf and root tissues; Ca-leaf/root, K-leaf/root, Mg-leaf/root, Fe-leaf/root, Mn-leaf/root indicate respective nutrient contents in leaf or root

To explore the systemic coordination among physiological, biochemical, and ionic traits in *S. officinalis* under Ni stress, Pearson correlation analysis and trait network mapping were conducted (Fig. 7). The resulting network consisted of 23 nodes and a dense set of positive and negative correlations (edges), forming two major trait association clusters. The first cluster linked biomass traits (DW, FW) with Chl *b* and essential shoot nutrients (Ca, K, Mg, and Fe), forming a growth-nutrition-photosynthesis association pattern. Strong correlations, such as DW–Fe shoot (*r* = 0.851) and FW–K shoot (*r* = 0.825), illustrate close associations between nutrient status, photosynthetic capacity, and biomass under metal stress.

The second cluster represented a redox–antioxidant response pattern. Proline showing significant positive correlations with MDA (*r* = 0.746), H_2_O_2_ (*r* = 0.800), and enzymatic antioxidants, including SOD (*r* = 0.819), POD (*r* = 0.857), and CAT (*r* = 0.833), indicating coordinated activation of oxidative stress markers and antioxidant defenses. Strong inter-correlations among antioxidant enzymes, such as CAT-POD (*r* = 0.917) and CAT-SOD (*r* = 0.915), reflect tight co-variation within the antioxidant system. Notably, TSP exhibited inverse correlations with both proline (*r* = − 0.818) and MDA (*r* = − 0.793), suggesting an association between protein status and stress-related metabolite accumulation.

In addition, Ni concentrations in roots and shoots showed consistent negative correlations with multiple essential nutrients. Ni shoot showed strong inverse associations with Ca shoot (*r* = − 0.892), K shoot (*r* = − 0.875), Mg shoot (*r* = − 0.851), and Fe shoot (*r* = − 0.890). Similarly, Ni root was negatively correlated with Ca root (*r* = − 0.901) and Fe shoot (*r* = − 0.909). These antagonistic correlations collectively indicate Ni-induced disruption of ionic balance and nutrient relationships. Together, the correlation network highlights the co-occurrence of oxidative stress responses, nutrient imbalance, and growth-related traits under Ni stress in *S. officinalis*.

#### Random forest classification highlights context-dependent discriminators in response to DA, Ni, and their combination

**Fig. 8:**
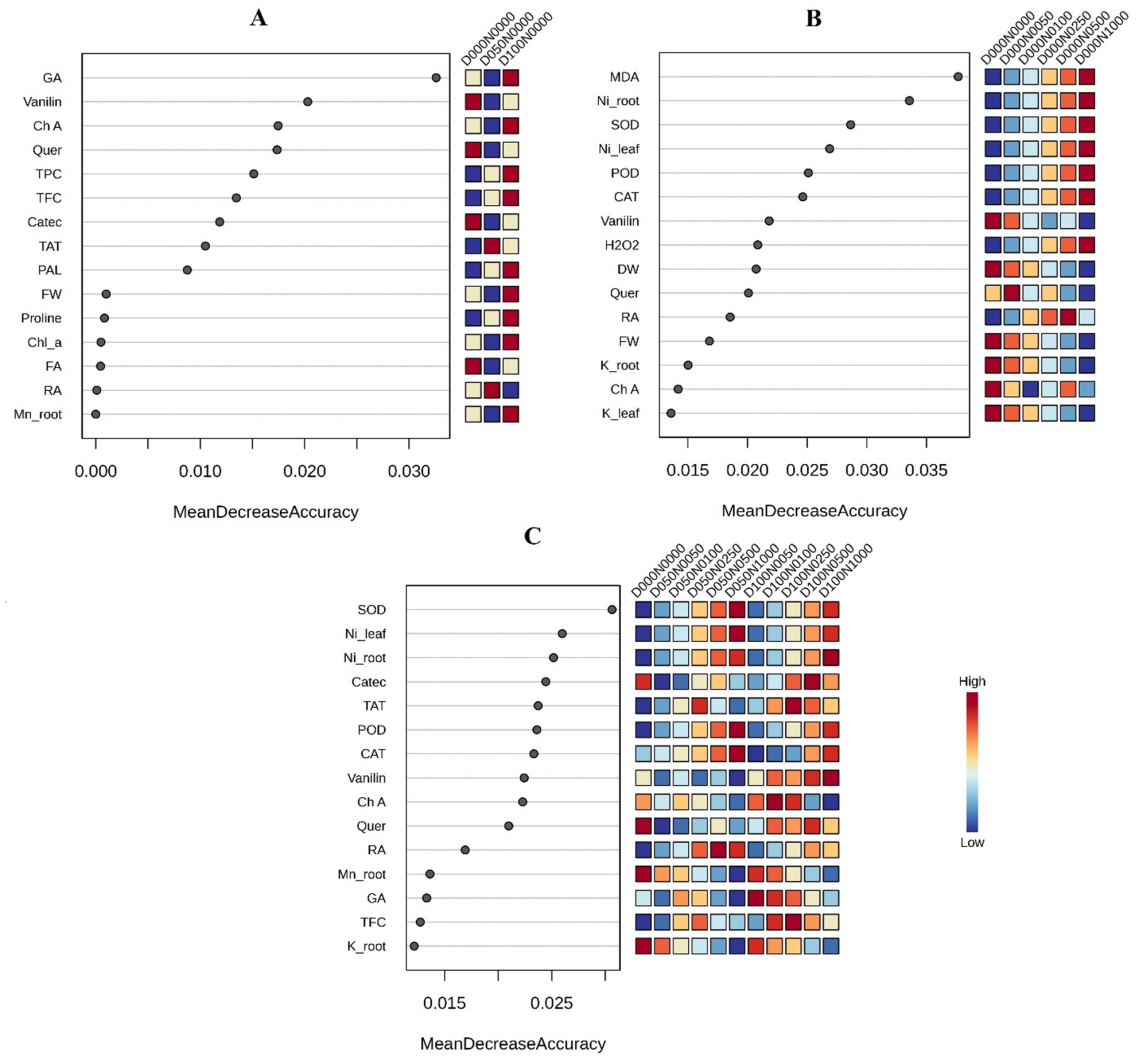
Random forest analysis highlighting the most informative traits driving treatment classification in *S. officinalis* under nickel (Ni) stress and dopamine (DA) supplementation. Variable importance (Mean Decrease Accuracy) is shown for (**A**) DA-specific classification, (**B**) Ni-specific classification, and (**C**) combined Ni–DA treatments. Adjacent heatmaps display scaled trait values, where red indicates higher and blue indicates lower levels relative to the mean. Key discriminating traits include phenolic compounds (e.g., GA, RA, Vanillin), antioxidant enzymes (e.g., SOD, CAT, POD), oxidative markers (e.g., MDA, H₂O₂), growth indices (FW, DW), and nutrient traits (e.g., Ni-root, Ni-leaf)

Exploratory RF models were constructed independently for DA application (Fig. 8A), Ni stress (Fig. 8B), and their combination (Fig. 8C) to qualitatively examine context-dependent patterns of trait contribution distinguishing treatment-specific responses in *S. officinalis*. These RF results are presented exclusively for pattern recognition and descriptive comparison and were not used to support mechanistic or inferential conclusions, given the limited sample size and high dimensionality of the dataset. In the DA-specific RF (Fig. 8A), phenolic-related traits showed higher relative contributions, with GA showing the largest MDA value (≈ 0.033), followed by vanillin (≈ 0.020), chlorogenic acid and quercetin (both ≈ 0.017), along with TPC and TFC.

In the Ni-specific RF model (Fig. 8B), higher MDA values were observed for oxidative stress-related parameters and Ni accumulation traits, including MDA (0.038), root Ni content (0.034), SOD (0.029), leaf Ni content (0.027), POD (0.025), and CAT (0.025). For the combined DA × Ni model (Fig. 8C), antioxidant enzymes and Ni-related traits exhibited comparatively higher MDA values, with SOD (0.031), leaf and root Ni contents, catechin, TAT, and POD (all > 0.024), while vanillin and chlorogenic acid (≈ 0.022) showed intermediate contributions.

Together, these exploratory RF models suggest context-dependent patterns in trait contribution: phenolic-related traits are more prominent under DA treatment, oxidative stress markers and Ni accumulation are more apparent under Ni stress, and a combined involvement of antioxidant enzymes, Ni content, and phenolic traits under the DA × Ni condition.

## Discussion

The present study aimed to investigate how *S. officinalis*, a stress-sensitive medicinal plant, responds to Ni toxicity and how DA modulates these responses. Although the highest Ni concentration (1000 µM) represents a severe stress condition and primarily induces phytotoxic effects, it was included to define the upper stress threshold and provide contextual boundaries for DA-mediated responses, complementing the more physiologically regulated responses observed at lower Ni concentrations. By integrating physiological, biochemical, ionic, and metabolic traits with multivariate analyses, we identified distinct modes of DA-mediated protection. The data suggest that DA not only attenuates the deleterious effects of Ni on plant growth and oxidative balance but also modulates stress adaptation strategies by coordinating the regulation of antioxidant systems and phenolic metabolism.

### Ni-induced pigment degradation and DA-mediated growth recovery

Excess Ni disrupted pigment biosynthesis and photosynthetic function in *S. officinalis*. Mechanistically, Ni impairs the tetrapyrrole and carotenoid pathways by inhibiting protochlorophyllide reductase and activating chlorophyllase, leading to pigment degradation and reduced carbon assimilation [3, 31]. Together with Ni-induced oxidative stress, these effects likely contribute to growth inhibition and biomass reduction.

Exogenous DA improved plant growth and pigment status in *S. officinalis*, particularly under moderate Ni stress. The protective effect of DA is likely associated with the stabilization of chloroplast structure and photosynthetic performance, including photosystem II functionality and Rubisco activity, as previously suggested under cadmium stress in legumes and aquatic plants [32, 33]. Together, these observations indicate that DA partially alleviates metal-induced photosynthetic impairment rather than fully restoring photosynthetic capacity.

### Oxidative stress induction under Ni toxicity and its attenuation by DA

Ni exposure induced a marked oxidative imbalance in *S. officinalis*, reflected by elevated H_2_O_2_ and MDA levels. These responses indicate disruption of redox homeostasis, potentially through interference with antioxidant signaling and membrane integrity. Although Ni is not inherently redox-active, it indirectly promotes oxidative stress by impairing cellular defense systems and enhancing ROS accumulation [34, 35]. DA application alleviated this oxidative burden by lowering H_2_O_2_ and MDA levels and improving redox balance in DA-treated plants [36].

Ni stress activated antioxidant enzyme systems in *S. officinalis*, reflecting engagement of primary ROS-scavenging pathways [37]. However, the persistence of elevated oxidative markers under severe Ni stress suggests that enzyme induction alone is insufficient under prolonged exposure to Ni [38]. DA treatment altered this oxidative profile by moderating antioxidant enzyme activities in parallel with reduced ROS levels, indicating that DA limits oxidative stress primarily by restricting ROS generation rather than by further intensifying enzymatic defenses [36]. This regulatory effect highlights DA as a modulator of redox homeostasis rather than a simple stress elicitor.

High Ni concentrations disrupted protein metabolism in *S. officinalis*, as reflected by reduced TSP levels, likely due to oxidative damage and impaired biosynthetic processes [39, 40]. Concurrently, proline levels increased markedly under severe Ni stress, consistent with its documented protective roles in osmotic regulation, ROS scavenging, and membrane stabilization in crops such as fenugreek and rice [6, 8, 41]. This stress-induced shift from protein synthesis toward compatible solute accumulation was partially reversed by DA, which promoted TSP recovery while moderating proline accumulation, reflecting reduced oxidative pressure rather than complete metabolic restoration. Together, these findings suggest that DA mitigates Ni-induced protein loss and restrains the overactivation of energy-intensive defense mechanisms, thereby supporting a more balanced metabolic state and cellular homeostasis under Ni stress.

The phenylpropanoid pathway, a central component of secondary metabolism, was activated in *S. officinalis* under Ni-induced oxidative stress, as reflected by increased TPC and TFC. These metabolites play multifunctional roles in metal stress tolerance, acting as metal chelators, ROS scavengers, and membrane stabilizers [42, 43], and similar responses have been reported in sweet potato under metal stress [44]. DA application further amplified this phenolic response, indicating a shift in antioxidant strategy toward phenol-based redox regulation. Support for this interpretation comes from DA-induced PAL activation and enhanced phenol biosynthesis reported in banana under cold stress [45]. These findings suggest that DA modulates phenylpropanoid metabolism to reinforce antioxidant capacity under Ni stress without invoking high metabolic costs.

The increase in phenolics under Ni stress is plausibly linked to activation of the phenylpropanoid pathway, with PAL acting as a key regulatory enzyme [46]. TAT, which channels tyrosine toward rosmarinic acid (RA) biosynthesis, may also contribute to phenolic modulation under stress [47]. While PAL activation in response to oxidative stress is well documented, the regulation of TAT under stress, particularly in relation to DA signaling, remains less explored [48]. The concurrent DA-associated enhancement of PAL and TAT therefore indicates a coordinated adjustment of phenolic metabolism, consistent with reports from other medicinal plants under abiotic stress, albeit in a species-dependent manner [48]. Collectively, these enzymatic responses provide a mechanistic basis for DA-related increases in total phenolics and flavonoids and may contribute to improved redox balance under Ni stress.

Ni-induced oxidative stress appears to differentially modulate phenolic metabolism in *S. officinalis*, with GA and RA exhibiting distinct response patterns. The biphasic behavior of GA under Ni exposure likely reflects its rapid utilization for early-phase ROS detoxification, followed by a compensatory upregulation at higher stress levels, consistent with its redox-active function [49]. Conversely, the steady accumulation of RA underscores its role as a more stable antioxidant reservoir, sustaining redox balance under prolonged oxidative conditions [17]. DA application further altered this phenolic balance by moderating GA accumulation while promoting RA biosynthesis. Such DA-associated modulation suggests a selective adjustment of phenolic metabolism toward compounds with greater antioxidant stability, potentially contributing to improved redox homeostasis under Ni stress.

### Ni-induced nutrient imbalance and its modulation by DA

Excess Ni appears to significantly disrupt the uptake and translocation of essential macro- and micronutrients in *S. officinalis*, with Ca and Fe being particularly affected. The impairment of Ca acquisition and mobility is consistent with reports in *Cicer arietinum* under metal stress [50]. Mechanistically, Ni^2+^ may disrupt Ca^2+^ and Fe^2+^ homeostasis through ionic competition for uptake pathways, partial entry via Ca^2+^ channels, and complex formation with chelators that restrict mobility and signaling [41, 51, 52]. Although Mg and K were also affected, their relatively stable TFs and Mn’s inconsistent response point to element-specific regulation; nevertheless, Mg depletion may exacerbate photosynthetic dysfunction due to its central role in chlorophyll structure [53]. Overall, these patterns support the notion that excess Ni disrupts divalent cation balance primarily through ionic competition [54]. While Ni treatments were applied at defined concentrations, interpretations of nutrient imbalance are primarily based on measured tissue Ni accumulation, as rhizosphere Ni availability was not directly quantified.

In this study, exogenous DA partially restored ionic balance under Ni stress, particularly by improving Ca and Fe uptake and translocation. These effects may be associated with DA-mediated attenuation of oxidative stress and preservation of membrane functionality, processes known to support nutrient transport under metal toxicity [55]. While Mg exhibited partial recovery, K and Mn responses were comparatively limited. DA application was also associated with a slight reduction in shoot Ni accumulation, potentially reflecting altered Ni distribution within plant tissues. Given the importance of vacuolar compartmentalization in heavy metal detoxification [56], DA-induced stabilization of plasma membrane integrity could contribute to reduced nonspecific Ni^2+^ influx while improving the selectivity and efficiency of divalent cation transport, particularly for Ca^2+^ and Fe^2+^, under Ni stress [55, 56].

### Advanced statistical analysis: multivariate insights into stress response coordination

Multivariate analyses highlighted the broad physiological disruptions caused by Ni toxicity in *S. officinalis*, while revealing DA’s capacity to partially reorient stress-related metabolism by modulating antioxidant and nutrient-linked pathways, rather than merely alleviating visible symptoms. PCA distinguished DA-treated samples under mild to moderate Ni stress, suggesting improved nutrient uptake and stabilization of pigment biosynthesis, both of which are linked through Fe- and Mg-dependent chlorophyll synthesis and membrane integrity [3, 32]. Under severe stress, however, DA’s protective influence waned, indicating a stress threshold beyond which homeostatic mechanisms collapse.

Heatmap clustering revealed partial separation between upstream regulators (PAL and TAT) and downstream phenolic metabolites, indicating differential regulation under Ni stress rather than complete dissociation. A similar separation was seen in wheat exposed to heavy metals [57]. This may indicate a way for the plant to adapt by shifting carbon during times of oxidative stress. Importantly, the samples treated with DA clustered with low-stress phenotypes, highlighting its role in restoring phenylpropanoid flux and encouraging the selective synthesis of high-value phenols.

Network analysis revealed two major trait association patterns: a nutrient-photosynthesis axis involving Fe, Ca, and Chl *b*, and a redox-related defense pattern comprising proline and antioxidant enzymes. DA effectively reduces proline and H_2_O_2_ levels [36], suggesting an association with lower ROS accumulation. The inverse relationship between TSP and proline/MDA suggests that DA may help maintain protein status under stress conditions.

RF analysis suggested distinct context-dependent patterns for DA and Ni responses. In DA-treated plants, phenolic compounds, especially gallic acid and vanillin, showed higher relative contributions, supporting the interpretation that DA is associated with enhanced phenylpropanoid metabolism and phenolic antioxidant production [58]. Ni stress, in contrast, was primarily characterized by oxidative damage (MDA) and metal accumulation, consistent with its known effects on ROS generation and nutrient imbalance [3, 4].

Under combined DA-Ni conditions, antioxidant enzymes such as SOD and POD showed increased relative activity, suggesting that DA may mitigate stress by reinforcing enzymatic ROS detoxification while sustaining phenolic metabolism [36].

## Conclusion

This study demonstrates that DA alleviates Ni-induced toxicity in *S. officinalis* by modulating redox homeostasis, improving nutrient balance, and enhancing antioxidant defenses. DA reduced oxidative damage, stabilized photosynthetic pigments, and promoted Ca and Fe uptake, while shifting defense strategies from enzyme-based responses toward phenolic-driven regulation, as evidenced by increased PAL and TAT activity and RA accumulation. Multivariate analyses supported DA’s role in linking nutrient–photosynthesis and redox-defence modules, underscoring its ability to promote an energy-efficient, phenolic-centred adaptive response under controlled conditions. These results suggest that DA may represent a promising candidate for mitigating heavy metal stress in medicinal plants, with potential relevance to sustainable cultivation and phytoremediation contexts, strategies pending further validation.

## Supplementary Information

Supplementary Material 1.

## Data Availability

The datasets generated and analyzed during the current study are available from the corresponding author upon reasonable request.
