# Supplementary material for: Dopamine modulates antioxidant and phenolic responses to alleviate nickel stress in Salvia officinalis
Source: BMC Plant Biol. 2026 Feb 11;26:491. doi: 10.1186/s12870-026-08365-5 (PMC12997738; doi:10.1186/s12870-026-08365-5)
Supplement: Supplementary file 1 — Supplementary Material 1. [file 12870_2026_8365_MOESM1_ESM.docx]

**Supplementary Table S1**. Two-way ANOVA showing the main and interactive effects of dopamine (DA) and nickel (Ni) on physiological, biochemical, antioxidant, and phenolic traits of Salvia officinalis. Significant effects (p < 0.05) are indicated by an asterisk (*). Abbreviations: FW, fresh weight; DW, dry weight; Chl a, chlorophyll a; Chl b, chlorophyll b; Car, carotenoids; H_2_O_2_, hydrogen peroxide; MDA, malondialdehyde; TSP, total slouble protein; SOD, superoxide dismutase; CAT, catalase; POD, peroxidase; PAL, phenylalanine ammonia-lyase; TAT, tyrosine aminotransferase; TPC, total phenolic content; TFC, total flavonoid content.

| **Variable** | **Source** | **F-value** | **p-value (Sig.)** | **Partial η^2^** | **Significance** |
| --- | --- | --- | --- | --- | --- |
| FW | Dopamine | 18.968 | 0.000 | 0.513 | * |
|  | Nickel | 40.643 | 0.000 | 0.850 | * |
|  | Dopamine * Nickel | 2.118 | 0.049 | 0.370 | * |
| DW | Dopamine | 13.885 | 0.000 | 0.435 | * |
|  | Nickel | 35.126 | 0.000 | 0.830 | * |
|  | Dopamine * Nickel | 1.677 | 0.125 | 0.318 |  |
| Chl a | Dopamine | 0.455 | 0.638 | 0.025 |  |
|  | Nickel | 0.835 | 0.533 | 0.104 |  |
|  | Dopamine * Nickel | 0.170 | 0.997 | 0.045 |  |
| Chl b | Dopamine | 38.544 | 0.000 | 0.682 | * |
|  | Nickel | 70.545 | 0.000 | 0.907 | * |
|  | Dopamine * Nickel | 1.517 | 0.174 | 0.296 |  |
| Car | Dopamine | 5.658 | 0.007 | 0.239 | * |
|  | Nickel | 11.348 | 0.000 | 0.612 | * |
|  | Dopamine * Nickel | 2.525 | 0.020 | 0.412 | * |
| TPC | Dopamine | 23.152 | 0.000 | 0.563 | * |
|  | Nickel | 35.812 | 0.000 | 0.833 | * |
|  | Dopamine * Nickel | 1.148 | 0.356 | 0.242 |  |
| TFC | Dopamine | 23.152 | 0.000 | 0.563 | * |
|  | Nickel | 35.812 | 0.000 | 0.833 | * |
|  | Dopamine * Nickel | 1.148 | 0.356 | 0.242 |  |
| Ca-leaf | Dopamine | 3.989 | 0.027 | 0.181 | * |
|  | Nickel | 55.332 | 0.000 | 0.885 | * |
|  | Dopamine * Nickel | 1.923 | 0.074 | 0.348 |  |
| K-leaf | Dopamine | 14.410 | 0.000 | 0.445 | * |
|  | Nickel | 79.817 | 0.000 | 0.917 | * |
|  | Dopamine * Nickel | 1.564 | 0.158 | 0.303 |  |
| Fe-leaf | Dopamine | 19.884 | 0.000 | 0.525 | * |
|  | Nickel | 110.893 | 0.000 | 0.939 | * |
|  | Dopamine * Nickel | 1.761 | 0.104 | 0.328 |  |
| Mg-leaf | Dopamine | 10.401 | 0.000 | 0.366 | * |
|  | Nickel | 39.345 | 0.000 | 0.845 | * |
|  | Dopamine * Nickel | 1.649 | 0.132 | 0.314 |  |
| Mn-leaf | Dopamine | 25.678 | 0.000 | 0.588 | * |
|  | Nickel | 53.585 | 0.000 | 0.882 | * |
|  | Dopamine * Nickel | 0.751 | 0.673 | 0.173 |  |
| Ni-leaf | Dopamine | 14.522 | 0.000 | 0.447 | * |
|  | Nickel | 335.852 | 0.000 | 0.979 | * |
|  | Dopamine * Nickel | 2.405 | 0.026 | 0.401 | * |
| Ca-root | Dopamine | 10.401 | 0.000 | 0.366 | * |
|  | Nickel | 39.345 | 0.000 | 0.845 | * |
|  | Dopamine * Nickel | 1.649 | 0.132 | 0.314 |  |
| K-root | Dopamine | 16.328 | 0.000 | 0.476 | * |
|  | Nickel | 74.745 | 0.000 | 0.912 | * |
|  | Dopamine * Nickel | 1.944 | 0.071 | 0.351 |  |
| Fe-root | Dopamine | 2.222 | 0.123 | 0.110 |  |
|  | Nickel | 37.452 | 0.000 | 0.839 | * |
|  | Dopamine * Nickel | 0.314 | 0.973 | 0.080 |  |
| Mg-root | Dopamine | 10.401 | 0.000 | 0.366 | * |
|  | Nickel | 39.345 | 0.000 | 0.845 | * |
|  | Dopamine * Nickel | 1.649 | 0.132 | 0.314 |  |
| Mn-root | Dopamine | 31.902 | 0.000 | 0.639 | * |
|  | Nickel | 128.395 | 0.000 | 0.947 | * |
|  | Dopamine * Nickel | 0.905 | 0.539 | 0.201 |  |
| Ni-root | Dopamine | 14.522 | 0.000 | 0.447 | * |
|  | Nickel | 335.852 | 0.000 | 0.979 | * |
|  | Dopamine * Nickel | 2.405 | 0.026 | 0.401 | * |
| H_2_O_2_ | Dopamine | 4.603 | 0.017 | 0.204 | * |
|  | Nickel | 49.036 | 0.000 | 0.872 | * |
|  | Dopamine * Nickel | 0.559 | 0.836 | 0.134 |  |
| MDA | Dopamine | 3.134 | 0.056 | 0.148 | * |
|  | Nickel | 29.658 | 0.000 | 0.805 | * |
|  | Dopamine * Nickel | 0.229 | 0.991 | 0.060 |  |
| TSP | Dopamine | 12.521 | 0.000 | 0.410 | * |
|  | Nickel | 32.607 | 0.000 | 0.819 | * |
|  | Dopamine * Nickel | 1.009 | 0.455 | 0.219 |  |
| POD | Dopamine | 21.144 | 0.000 | 0.540 | * |
|  | Nickel | 307.512 | 0.000 | 0.977 | * |
|  | Dopamine * Nickel | 2.117 | 0.049 | 0.370 | * |
| SOD | Dopamine | 22.223 | 0.000 | 0.552 | * |
|  | Nickel | 313.351 | 0.000 | 0.978 | * |
|  | Dopamine * Nickel | 1.351 | 0.242 | 0.273 |  |
| CAT | Dopamine | 44.874 | 0.000 | 0.714 | * |
|  | Nickel | 371.931 | 0.000 | 0.981 | * |
|  | Dopamine * Nickel | 4.613 | 0.000 | 0.562 | * |
| Proline | Dopamine | 4.680 | 0.016 | 0.206 | * |
|  | Nickel | 28.180 | 0.000 | 0.796 | * |
|  | Dopamine * Nickel | 1.886 | 0.080 | 0.344 |  |
| PAL | Dopamine | 4.431 | 0.019 | 0.198 | * |
|  | Nickel | 10.175 | 0.000 | 0.586 | * |
|  | Dopamine * Nickel | 1.814 | 0.093 | 0.335 |  |
| TAT | Dopamine | 83.648 | 0.000 | 0.823 | * |
|  | Nickel | 56.891 | 0.000 | 0.888 | * |
|  | Dopamine * Nickel | 1.255 | 0.292 | 0.259 |  |
| Gallic acid | Dopamine | 81.037 | 0.000 | 0.818 | * |
|  | Nickel | 11.545 | 0.000 | 0.616 | * |
|  | Dopamine * Nickel | 17.950 | 0.000 | 0.833 | * |
| Cathechin | Dopamine | 53.572 | 0.000 | 0.749 | * |
|  | Nickel | 13.277 | 0.000 | 0.648 | * |
|  | Dopamine * Nickel | 10.033 | 0.000 | 0.736 | * |
| Quersetin | Dopamine | 103.610 | 0.000 | 0.852 | * |
|  | Nickel | 6.392 | 0.000 | 0.470 | * |
|  | Dopamine * Nickel | 11.911 | 0.000 | 0.768 | * |
| Vanilin | Dopamine | 62.174 | 0.000 | 0.775 | * |
|  | Nickel | 3.028 | 0.022 | 0.296 | * |
|  | Dopamine * Nickel | 7.857 | 0.000 | 0.686 | * |
| Ferulic acid | Dopamine | 1.135 | 0.333 | 0.059 |  |
|  | Nickel | 1.209 | 0.325 | 0.144 |  |
|  | Dopamine * Nickel | 0.805 | 0.625 | 0.183 |  |
| Rosmarinic acid | Dopamine | 95.993 | 0.000 | 0.842 | * |
|  | Nickel | 76.574 | 0.000 | 0.914 | * |
|  | Dopamine * Nickel | 5.743 | 0.000 | 0.615 | * |
| Chlorogenic acid | Dopamine | 46.079 | 0.000 | 0.719 | * |
|  | Nickel | 13.653 | 0.000 | 0.655 | * |
|  | Dopamine * Nickel | 14.115 | 0.000 | 0.797 | * |
